# Generation and characterization of a novel knockin minipig model of Hutchinson-Gilford progeria syndrome

**DOI:** 10.1038/s41421-019-0084-z

**Published:** 2019-03-19

**Authors:** Beatriz Dorado, Gro Grunnet Pløen, Ana Barettino, Alvaro Macías, Pilar Gonzalo, María Jesús Andrés-Manzano, Cristina González-Gómez, Carlos Galán-Arriola, José Manuel Alfonso, Manuel Lobo, Gonzalo J. López-Martín, Antonio Molina, Raúl Sánchez-Sánchez, Joaquín Gadea, Javier Sánchez-González, Ying Liu, Henrik Callesen, David Filgueiras-Rama, Borja Ibáñez, Charlotte Brandt Sørensen, Vicente Andrés

**Affiliations:** 1 Centro Nacional de Investigaciones Cardiovasculares (CNIC), 28029 Madrid, Spain; 2 CIBER en Enfermedades Cardiovasculares (CIBER-CV), Madrid, Spain; 3 Department of Clinical Medicine, Aarhus University, 8200 Aarhus, Denmark; 4 Department of Cardiology, Aarhus University Hospital, 8200 Aarhus, Denmark; 5 Laboratory of Physiology and Biotechnology of Reproduction in Swine, INIA (Spanish National Institute for Agricultural and Food Research and Technology), Madrid, Spain; 6 Department of Physiology, University of Murcia and IMIB-Arrixaca, 30100 Murcia, Spain; 7 Philips HealthCare Iberia, 28050 Madrid, Spain; 8 Department of Animal Science, Aarhus University, 8830 Tjele, Denmark; 9 Department of Cardiology, Cardiac Electrophysiology Unit, Hospital Clínico San Carlos, Madrid, Spain; 10 Department of Cardiology, Instituto de Investigación Sanitaria-Fundación Jiménez Díaz Hospital, Madrid, Spain

**Keywords:** Molecular biology, Nuclear organization

## Abstract

Hutchinson-Gilford progeria syndrome (HGPS) is an extremely rare genetic disorder for which no cure exists. The disease is characterized by premature aging and inevitable death in adolescence due to cardiovascular complications. Most HGPS patients carry a heterozygous de novo *LMNA* c.1824C > T mutation, which provokes the expression of a dominant-negative mutant protein called progerin. Therapies proven effective in HGPS-like mouse models have yielded only modest benefit in HGPS clinical trials. To overcome the gap between HGPS mouse models and patients, we have generated by CRISPR-Cas9 gene editing the first large animal model for HGPS, a knockin heterozygous *LMNA* c.1824C > T Yucatan minipig. Like HGPS patients, HGPS minipigs endogenously co-express progerin and normal lamin A/C, and exhibit severe growth retardation, lipodystrophy, skin and bone alterations, cardiovascular disease, and die around puberty. Remarkably, the HGPS minipigs recapitulate critical cardiovascular alterations seen in patients, such as left ventricular diastolic dysfunction, altered cardiac electrical activity, and loss of vascular smooth muscle cells. Our analysis also revealed reduced myocardial perfusion due to microvascular damage and myocardial interstitial fibrosis, previously undescribed readouts potentially useful for monitoring disease progression in patients. The HGPS minipigs provide an appropriate preclinical model in which to test human-size interventional devices and optimize candidate therapies before advancing to clinical trials, thus accelerating the development of effective applications for HGPS patients.

## Introduction

Hutchinson-Gilford progeria syndrome (HGPS) is an extremely rare disorder (prevalence of 1 in 20 million; https://www.progeriaresearch.org/) characterized by premature aging and death during adolescence^1,2^. “Classical” HGPS is caused by a de novo heterozygous mutation in the *LMNA* gene (encoding A-type lamins), with more than 90% of patients carrying a c.1824C > T (pG608G) point mutation^3,4^. This mutation activates usage of an alternative 5′ splice donor site in exon 11 that results in deletion of 150 nucleotides from *LMNA* mRNA and the synthesis of a truncated protein called progerin. This aberrant protein accumulates in the nuclear envelope due to irreversible farnesylation and causes severe alterations in multiple cellular functions^1,2^ (Supplementary Fig. S1).

HGPS patients appear normal at birth and typically do not manifest signs of disease until around 1–2 years of age, when they begin to exhibit failure to thrive and develop symptoms reminiscent of physiological aging, including alopecia, lipodystrophy, pigmented spots and skin wrinkling with sclerodermia, and bone-skeletal dysplasia. One of the main alterations in HGPS is cardiovascular disease (CVD), featuring atherosclerosis, vascular stiffening and calcification, electrocardiographic (ECG) alterations, and left ventricular (LV) diastolic dysfunction^5–9^. To date, there is no effective therapy or cure for HGPS, and patients die at an average age of 14.6 years predominantly due to CVD complications^10^.

The extreme rarity of HGPS makes the organization of any clinical trial a huge challenge where the inevitable limitation of a small patient cohort adds to the difficulty of deciding which therapies effective in HGPS-like mice should be tested in patients. Available HGPS mouse models either ectopically express progerin, lack or overexpress A-type lamin isoforms, or accumulate farnesylated prelamin A (Supplementary Fig. S1)^2,11^. Despite their limitations, HGPS-like mice have been the gold-standard preclinical model and have led to clinical trials testing the ability of repurposed drugs to reduce progerin farnesylation^12^. Targeting progerin farnesylation resulted in a mild benefit in body weight, bone, and vascular alterations in a subset of HGPS patients and was associated with lower mortality rate after 2.2 years of follow-up; however, the estimated increase in life expectancy is only 1.6 years^10,13–15^, highlighting the limitations in translating results of preclinical mouse studies to HGPS patients.

New gene editing methodologies are enabling translational biomedicine to bridge the gap between mice and humans through the use of pig models^16–20^. Pigs share strong genetic, anatomical, and physiological similarities with humans, and they are increasingly used for preclinical testing of preventive or therapeutic drugs and other interventions, toxicity tests, studies of human disease processes, and functional genomics^21,22^. Particularly relevant to HGPS is the close similarity of the pig and human cardiovascular systems; pig and human hearts have a similar size and, together with primates, the pig model provides the closest match to human coronary vasculature, blood flow, hemodynamics, and myocardial contractility. Indeed, the growth of the heart and vascular system in pigs from birth to 4 months of age is analogous to the growth of the same system in humans into the mid-teens^23^. Lipoprotein profiles and metabolism are also very similar in pigs and humans.

Yucatan minipigs are sociable, reach puberty at around 4–6 months of age, have a life expectancy of ~10 years, and can grow to ~80kg^24^. Yucatan minipigs are therefore manageable throughout their life, while being large enough for study or intervention with the same technology used for human diagnosis. To overcome the gap between progeroid mouse models and HGPS patients, here we describe the generation and characterization of the first pig model of “classical” HGPS, a knockin Yucatan minipig harboring the heterozygous *LMNA* c.1824C > T mutation. Like patients, HGPS minipigs endogenously co-express progerin and A-type lamins and recapitulate all the main symptoms of human HGPS, including CVD and its death-related complications.

## Results

### Generation of HGPS Yucatan minipigs

**Fig. 1:**
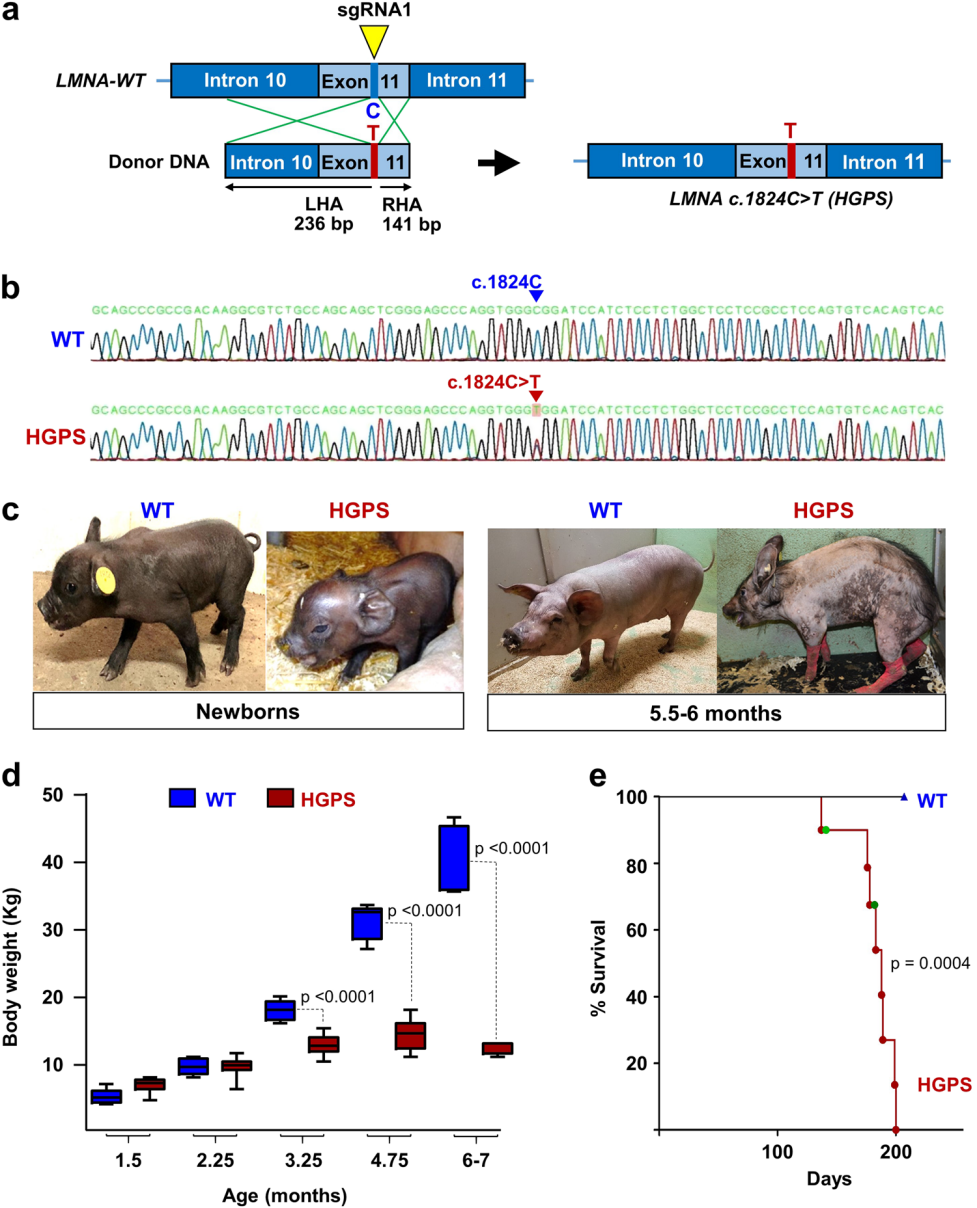
Knockin heterozygous LMNA c.1824C > T Yucatan minipigs exhibit HGPS-like external phenotype, growth retardation, and premature death. **a** Genetic engineering procedure for generating Hutchinson-Gilford progeria syndrome (HGPS) knockin Yucatan minipig fibroblasts by CRISPR/Cas9-mediated homologous recombination. The area of hybridization for sgRNA1 in genomic DNA is indicated by a yellow arrowhead, the area of recombination between genomic DNA and donor DNA template is indicated by green lines, and the single C > T base mutation in the donor DNA template is indicated by a red T in exon 11. LHA and RHA respectively indicate the left and right homology arms surrounding the C > T mutation (not drawn to scale). For an overview of the whole method to generate HGPS minipigs and its efficiency see Supplementary Fig. S2. For detailed information of molecular tools see Supplementary Table S1 and 2. **b** Genomic *LMNA* exon 11 sequence of wild-type (WT) minipig fibroblasts and the generated heterozygous HGPS knockin fibroblast clone. Note that WT cells are homozygous for the C nucleotide in position c.1824 (blue arrowhead), and HGPS cells harbor both the WT C allele and the mutant T allele (red arrowhead). **c** Representative photographs showing the normal appearance of HGPS minipigs at birth and the severe premature-aging external phenotype at 5.5–6 months of age. Note that pictures were not taken at the same distance. See also Supplementary Fig. S3 and Movies S1-5. **d** HGPS Yucatan minipigs experience difficulty to thrive. The graph shows body weight/age curves (*n* = 6 WT; *n* = 10 HGPS). **e** HGPS minipigs die prematurely. Green dots in the Kaplan-Meier survival curve are censored data (1 HGPS minipig died during anesthesia induction in preparation for imaging tests and another was euthanized due to humane end-point criteria)

HGPS knockin Yucatan minipigs carrying a heterozygous *LMNA* c.1824C > T mutation were generated using CRISPR (clustered regularly interspaced short palindromic repeats)-Cas9 (CRISPR-associated protein 9) gene editing^25,26^ in male Yucatan minipig skin fibroblasts, followed by somatic cell nuclear transfer (SCNT) by handmade cloning to enucleated oocytes from Large White sows^27^ (Supplementary Fig. 2a). Our genetic engineering strategy omitted insertion of antibiotic or fluorescent selection cassettes in the genomic DNA while retaining the intact PAM sequence for Cas9 guidance to avoid the generation of potential splicing sites in addition to the one resulting from introduction of the *LMNA* c.1824C > T mutation. Three single guide RNAs (sgRNAs) were constructed to target exon 11 in the porcine *LMNA* gene. sgRNA1 was selected for subsequent gene editing experiments due to its consistency when tested in an in vitro validation assay^28^. Information on the different molecular tools is available in Supplementary Tables S1–2. Skin primary fibroblasts from a newborn male Yucatan minipig were co-transfected with sgRNA1, a Cas9 plasmid construct, an enhanced green fluorescent protein (EGFP) reporter plasmid, and a donor DNA construct including the c.1824C > T mutation flanked by a left and a right homology arm (see Fig. 1a for donor DNA template design and Supplementary Table S1 for the primers used for its generation). To enrich for transfected cells, EGFP-expressing cells were isolated by fluorescence-activated cell sorting, seeded on 96-well plates, and expanded as single-cell colonies. Allele-specific PCR was performed on lysates from all surviving cell clones to select c.1824C > T-positive clones. Subsequent sequencing identified only one clone harboring the desired heterozygous *LMNA* c.1824C > T mutation without unwanted insertions, deletions, or base changes in the region involved in homologous recombination (Fig. 1b). Moreover, this HGPS cell clone showed no evidence of off-target Cas9 effects or random integration of the vectors used (sgRNA, Cas9, EGFP-N3, and the donor molecule backbone). The clone was therefore used as the nuclear donor for SCNT into enucleated oocytes from Large White sows^27^. Viable reconstructed embryos were transferred to three surrogate Large White sows^29^. A total of 19 cloned male piglets were obtained (5 stillborn and 14 liveborn), of which 10 survived the postnatal period (Supplementary Fig. 2b). All obtained piglets were genotyped as heterozygous for *LMNA* c.1824C > T.

### Growth retardation, external symptoms, and premature death

The HGPS piglets had a normal appearance and size at birth (Fig. 1c, left; Supplementary Movie S1) and until ~2.5 months of age (Supplementary Movie S2). From then on, the HGPS minipigs progressively developed an angular and narrow face, dry and taut skin, occasional difficulty in standing up due to joint rigidity, and failure to thrive (Fig. 1d). From ~4 to 4.5 months of age, body weight gain was severely impaired (Fig. 1d). Moreover, from this age, all the HGPS minipigs showed frailty and symptoms reminiscent of human HGPS, with intensity varying between individuals. These symptoms included prominent eyes and short eyelids with frequent corneal ulcerations, wrinkled and taut skin with patchy punctate pigmentation, large areas of alopecia and/or areas with hardened/thickened hair, a sculpted nose, thin lips, prominent teeth, a below-normal body mass index accompanied by subcutaneous fat loss and prominent bones, and thin limbs with severe joint stiffness (Fig. 1c, right; Supplementary Fig. S3, and Movies S3-5).

None of the HGPS minipigs survived beyond the age of 6.6 months (survival ranging from 137 to 200 days; mean life span 177.3 ± 21.4 days) (Fig. 1e). One HGPS minipig was euthanized at 182 days of age due to humane end-point criteria, and another pig died at 141 days of age during anesthesia induction in preparation for in vivo cardiac function tests. Compared with age-matched wild-type (WT) controls, ~5- to 5.5-month-old HGPS minipigs showed no major differences in hematology and coagulation parameters (not shown). Likewise, serum analysis revealed no alterations in total cholesterol, free cholesterol, or triglycerides between WT and HGPS minipigs; however, HGPS minipigs had above-normal serum low-density lipoprotein and below-normal serum high-density lipoprotein (Supplementary Table S3), similar to HGPS patients^6^.

### Progerin expression

**Fig. 2:**
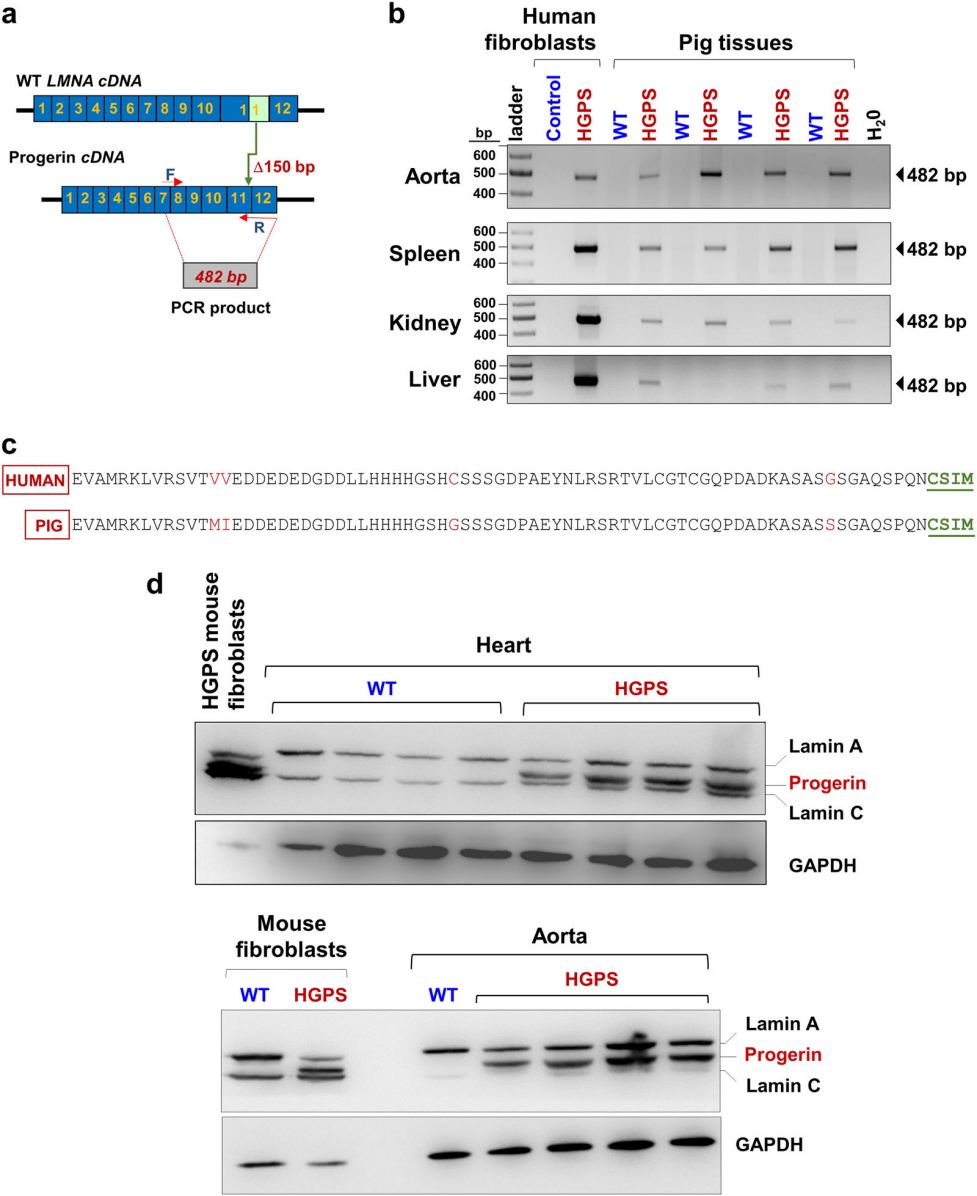
Progerin expression in Hutchinson-Gilford progeria syndrome (HGPS) minipigs. **a** Design of primers for PCR amplification of progerin mRNA. F forward primer, R reverse primer (which spans from progerin exon 12 to the 3′ end of exon 11 across the in-frame 150-nucleotide deletion produced by the alternative splicing caused by the *LMNA* c.1824C > T mutation). **b** Representative agarose gels showing PCR detection of progerin mRNA in wild-type (WT) and HGPS minipig tissues (each lane corresponds to a different pig). Skin fibroblasts from HGPS patients and healthy controls were used as positive and negative controls, respectively. First lane, DNA ladder. **c** Comparison of the C-terminal amino-acid sequences of human progerin and HGPS minipig progerin, which was predicted with ExPASy software from the DNA sequence of the gel-purified 482-bp PCR product specific to HGPS pigs (cf. **b**). **d** Representative immunoblots for lamin A/C and progerin protein in WT and HGPS minipig heart and aorta (heart: 60 µg protein/lane, aorta: 30 µg protein/lane; each lane corresponds to a different pig). Fibroblasts from WT and HGPS mice (*Lmna*^*G609G*^) were used as progerin expression-negative and -positive controls, respectively. GADPH was used as loading control

Primers for PCR-based detection of progerin mRNA in porcine tissues were based on the human progerin mRNA sequence (NCBI accession number NM_001282626.1) (Fig. 2a and Materials and methods). All HGPS minipig tissues tested were positive for a 482-bp band corresponding to an amplicon spanning a 150-nucleotide deletion caused by the LMNA 1824C > T mutation; this band was absent in WT tissues (Fig. 2b). Sequencing of this amplicon revealed 94.9% homology between the 3′ end of progerin mRNA (exon 10–12) in HGPS minipigs and HGPS patients, with only a few mismatches corresponding to single-nucleotide polymorphisms between the two species. Remarkably, the predicted amino-acid sequence corresponding to the HGPS pig 3′ mRNA revealed conservation of the critical C-terminal CSIM motif, which is irreversibly farnesylated and carboxy-methylated in human progerin (Fig. 2c). We also investigated progerin protein expression in heart and aorta, which are severely affected in HGPS. Western blot demonstrated the presence of progerin in HGPS tissues but not in WT tissues, with an electrophoretic mobility between those of lamin A and lamin C (Fig. 2d). These results demonstrate that heterozygous *LMNA* c.1824C > T Yucatan minipigs, like HGPS patients, endogenously co-express progerin mRNA and protein in all tissues tested.

### Myocardial microvascular dysfunction and fibrosis

**Fig. 3:**
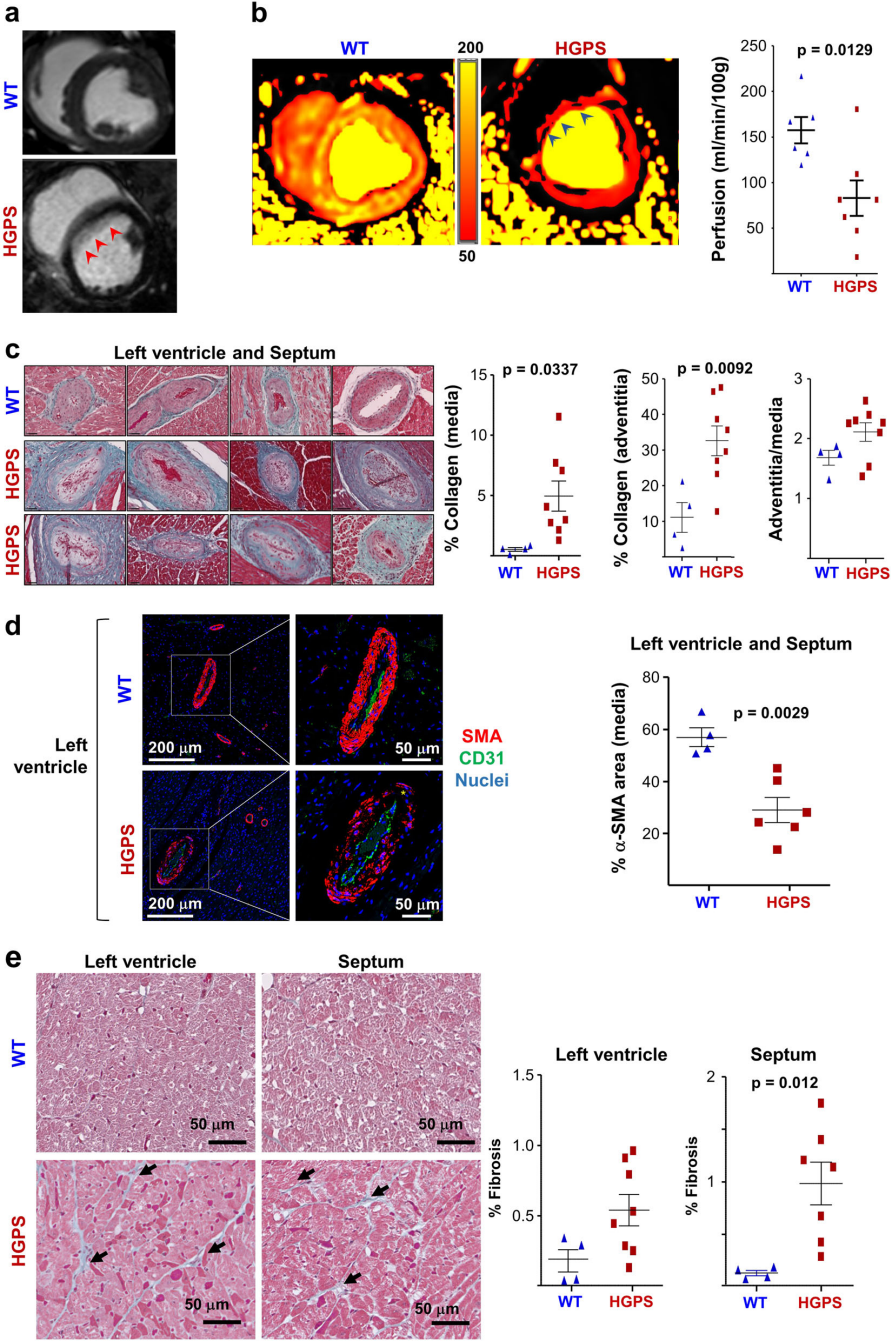
Heart microvascular dysfunction and fibrosis in Hutchinson-Gilford progeria syndrome (HGPS) minipigs. **a** Left ventricular (LV) fibrosis in HGPS minipigs revealed by delayed gadolinium enhancement magnetic resonance imaging (MRI). Representative images are shown. Red arrowheads indicate the area of contrast deposition. **b** LV microvascular damage in HGPS minipigs determined by cardiac MRI-determined absolute quantitative perfusion after gadolinium administration (*n* = 6 WT and *n* = 7 HGPS minipigs, 4.3–5.5 months old). The images show representative examples of LV flow with perfusion ranging from 50 (red) to 200 (yellow) ml/min/100 g. Arrowheads indicate the non-perfused area. **c** Collagen deposition in LV and septal coronary arteries of WT and HGPS hearts. Images show representative Masson trichrome (MT) staining of heart cross sections, revealing increased collagen deposition (green/blue staining) in HGPS hearts. Left and center graphs: mean percentage collagen content in medial and adventitial layers of arterioles >65 μm in diameter, quantified by image deconvolution (area analyzed, 170 mm^2^ in LV and 120 mm^2^ in septum). Right graph: adventitial hyperthrophy quantified as the ratio of adventitial to medial perimeter (11–27 arterioles of mean diameter >50 μm per minipig in LV or septum). **d** Smooth muscle cell loss in the medial layer of LV and septum arterioles in HGPS minipigs. Representative immunofluorescence images show staining for α-smooth muscle actin (SMA) in red, CD31 (endothelial cell marker) in green, and nuclei in blue. The graph shows mean percentage SMA signals in the medial layer of 12–41 arterioles per minipig in LV or septum. See also Supplementary Fig. S4. **e** Representative images of MT staining in heart cross sections, showing myocardial fibrosis in HGPS minipigs. Black arrows indicate areas of intense MT staining. Images were deconvoluted to quantify mean percentage myocardial fibrosis in vessel-free myocardial areas (seven areas in LV and six areas in septum; 470 μm^2^ each region analyzed)

We performed cardiac magnetic resonance imaging (MRI) in 4.3- to 5.4-month-old HGPS and WT Yucatan minipigs. T1 and T2 mapping and overall LV extracellular volume fraction showed no significant differences between WT and HGPS minipigs. However, qualitative analysis of late gadolinium contrast enhancement images revealed patchy gadolinium deposition in the anterior LV wall and septal regions in three out of eight HGPS minipigs (Fig. 3a). Cardiac MRI (cMRI)-quantified myocardial perfusion^30^ was significantly lower in HGPS minipigs (Fig. 3b), indicating overt LV microvascular damage.

We next performed hematoxilyn & eosin (H&E) and Masson trichrome (MT) staining in transverse heart sections (between the atrium and apex) obtained after necropsy. Compared with controls, the LV of the HGPS minipigs had double amount of medium and large coronary arteries (medium, 65–250 μm diameter; large, >250 μm diameter, including the medial layer). Moreover, in five out of eight HGPS minipigs, ~21% of these vessels showed signs of atherosclerotic disease including medial degeneration and intimal hypertrophy (Supplementary Fig. S4a, b). The amounts of medium/large coronaries in the septum were similar in WT and HGPS minipigs, but more than 21% of these vessels were degenerated in three out of eight HGPS minipigs (occlusion ranging from 26 to 83% of coronaries in different HGPS minipigs) (Supplementary Fig. S4a–b). In addition, MT staining in medium/large LV and septal coronaries revealed significantly higher collagen deposition in both the medial and adventitial layers of arterioles in HGPS minipigs (Fig. 3c), accompanied by a trend toward adventitial hypertrophy in six out of eight HGPS minipigs (Fig. 3c, ratio adventitia/media). We also observed occasional vessel occlusion in HGPS minipigs in the subpleural arteries, aorta, and vasa vasorum (not shown).

Loss of smooth muscle cells is a hallmark of human and mouse HGPS vessels^31–35^. Consistent with these findings, immunofluorescence studies demonstrated significantly lower α-smooth muscle actin immunoreactivity in the medial layer of medium/large LV and septal coronaries in HGPS minipigs (Fig. 3d and Supplementary Fig. S4c).

Although H&E staining did not reveal gross myocardial tissue abnormalities, image quantification after MT deconvolution demonstrated significantly higher interstitial fibrosis in the septum of HGPS minipigs than in similar locations in WT minipig hearts (mean % of six vessel-free areas of 470 μm^2^ each) (Fig. 3e). Together, these histological results confirm the in vivo MRI findings, demonstrating increased myocardial fibrosis and reduced perfusion (cf. Fig. 3a, b).

### Systolic and diastolic myocardial dysfunction

**Fig. 4:**
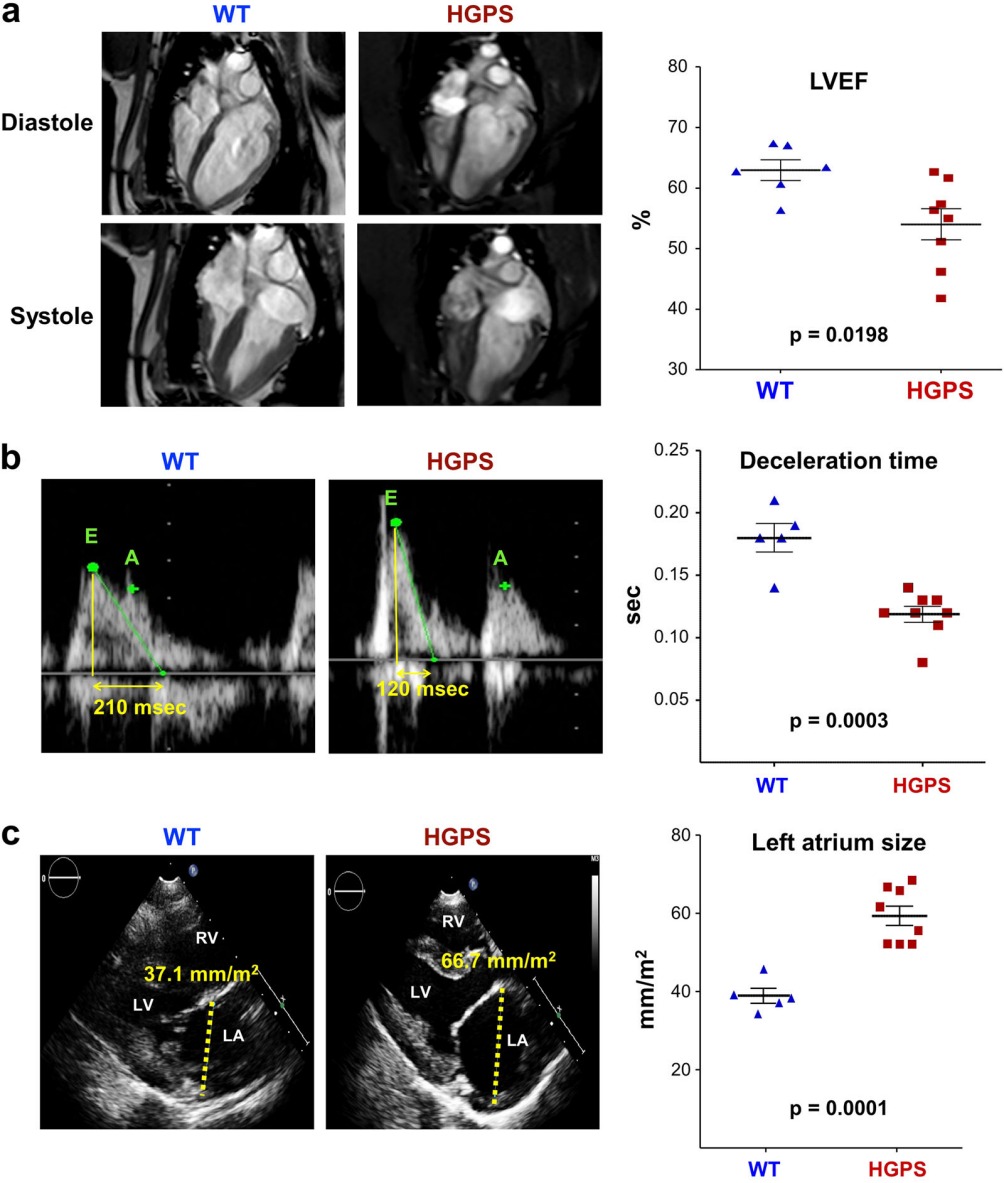
Systolic and diastolic dysfunction in Hutchinson-Gilford progeria syndrome (HGPS) minipigs. Cardiac magnetic resonance imaging (MRI) and echocardiography analysis of HGPS minipigs (*n* = 7–8) and wild-type (WT) minipigs (*n* = 5–6) of a similar age (~4.3–5.5 months) revealed enlarged left atria (LA) and increased left ventricular (LV) filling pressures, as well as systolic and diastolic LV dysfunction in HGPS minipigs. **a** MRI measurements of LV ejection fraction (LVEF). Representative four chamber long axis views are shown on the left. **b** Echocardiographic measurements of mitral early wave deceleration time (shown in yellow numbers in the representative images on the left). **c** Echocardiographic measurements of LA size (shown in yellow numbers in the representative images on the left. See also unaffected systolic and diastolic parameters in HGPS minipigs in Supplementary Fig. S5

We examined cardiac function in 4.3- to 5.4-month-old HGPS and WT Yucatan minipigs using gold-standard clinical non-invasive techniques, including cardiac MRI and transthoracic echocardiography. MRI examination revealed no significant between-genotype differences in LV size (measured as LV end-diastolic volume: LVEDV) or LV mass (Supplementary Fig. S5a–b). However, the HGPS minipigs displayed overt LV systolic dysfunction, with a significantly lower LV ejection fraction (LVEF) than controls (Fig. 4a). Echocardiography revealed diastolic dysfunction in HGPS minipigs, evidenced by several mitral inflow Doppler parameters, including a trend to lower *E*′ wave, and higher *E*/*E*′ ratios (Supplementary Fig. S5c-d) and a significantly shorter early mitral deceleration time (DT) (Fig. 4b). In agreement with the Doppler findings, the HGPS minipigs had significantly enlarged left atria (Fig. 4c). Moreover, two out of eight HGPS minipigs showed signs of mild mitral valve degeneration with accompanying mitral regurgitation. In contrast, the aortic, pulmonary, and tricuspid valves showed no alterations in HGPS minipigs. Overall, cardiac phenotyping by advanced multimodality imaging showed signs of mixed systolic and diastolic heart dysfunction in the HGPS minipigs.

### ECG alterations and severe pre-mortem cardiac conduction abnormalities

**Fig. 5:**
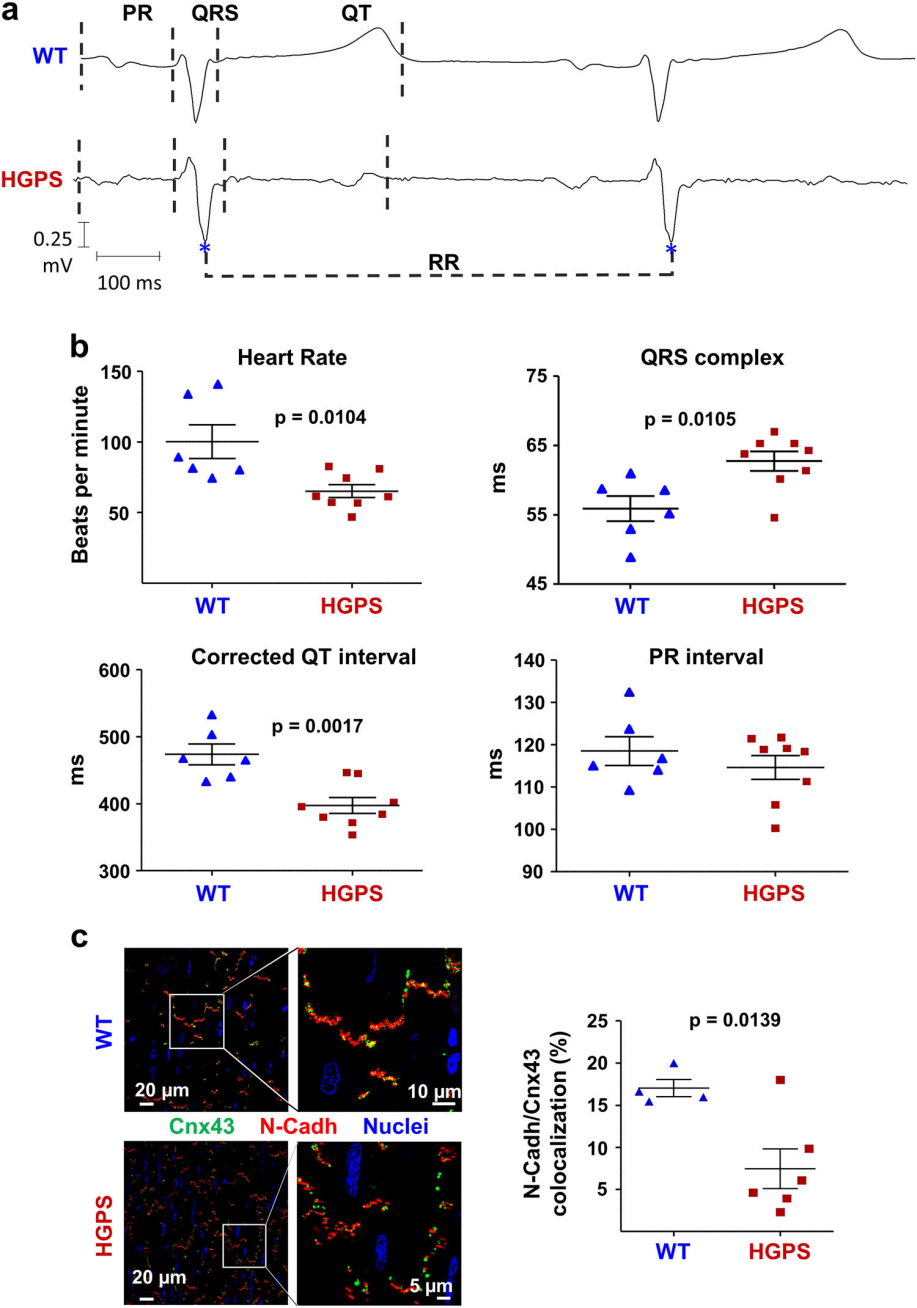
Electrocardiographic (ECG) alterations and aberrant connexin 43 (Cnx43) localization in the hearts of Hutchinson-Gilford progeria syndrome (HGPS) minipigs. **a** Representative ECG traces recorded in wild-type (WT) and HGPS minipigs. **b** HGPS minipigs show bradycardia and QRS complex prolongation on ECG. For each parameter, a set of 10 beats per animal was analyzed and the mean was represented. **c** Cnx43 is mislocalized in the plasma membrane of HGPS cardiomyocytes. Representative immunofluorescence images of heart tissue show staining for Cnx43 (green), N-Cadh (red), and nuclei (blue). The graph shows the percentage of N-Cadh/Cnx43 co-localization based on the mean of three 236 µm^2^ areas per heart tissue sample

We next recorded 12-lead ECG tracings (Fig. 5a, b). The HGPS minipigs had a significantly lower heart rate and a wider QRS complex than WT controls. Conversely, corrected QT intervals were shorter in the HGPS minipigs, with no significant changes in the PR interval.

We also performed confocal microscopy studies in heart to investigate the localization of connexin 43 (Cnx43) and N-Cadherin (N-Cadh); these proteins are normally expressed in the intercalated disk between adjacent cardiomyocytes and are essential for proper mechanical and electrical coupling and propagation of the electrical impulse^36^. The cardiomyocyte membranes of HGPS minipigs showed aberrant Cnx43 localization associated with below-normal N-Cadh/Cnx43 co-localization (Fig. 5c).

**Fig. 6:**
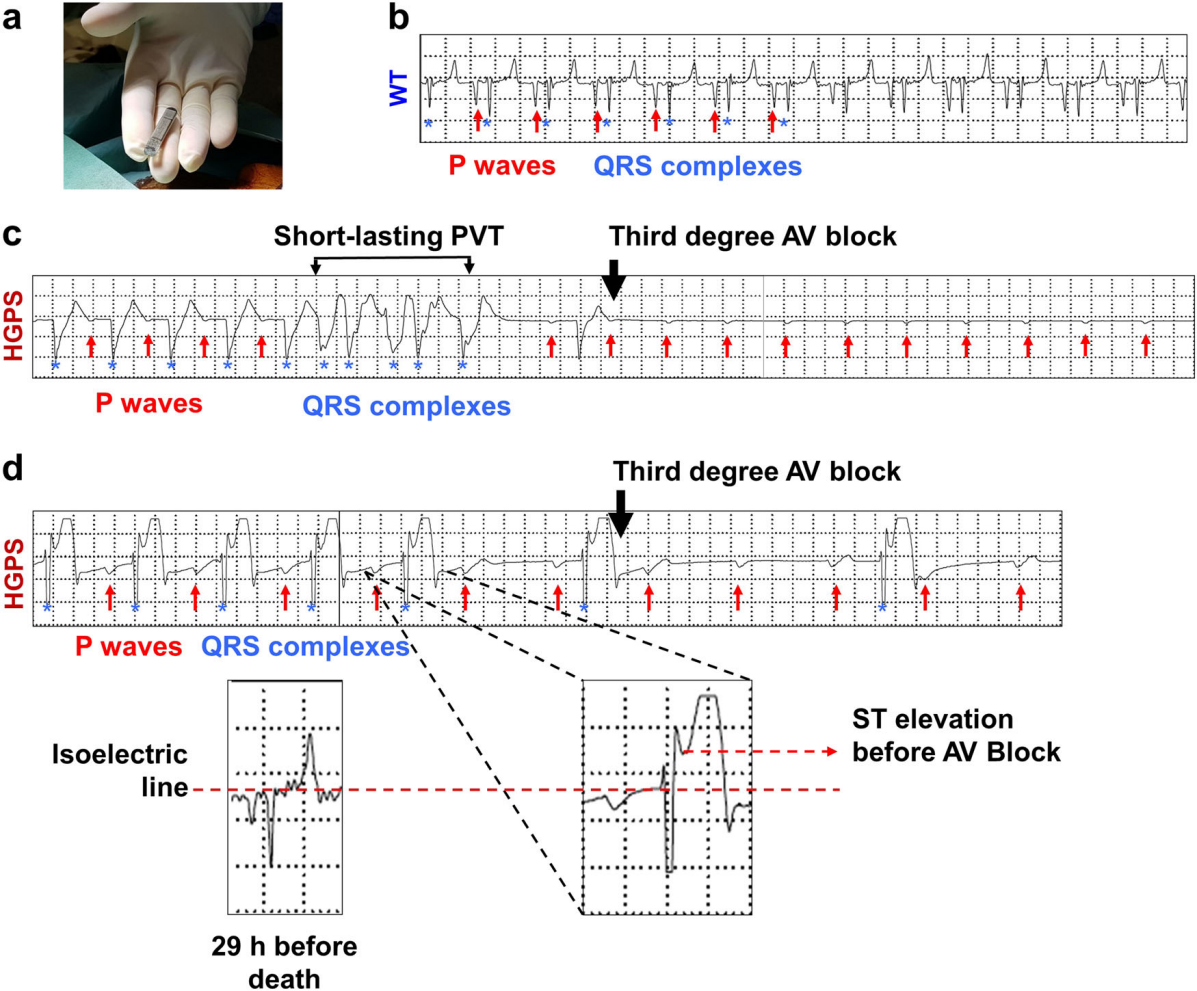
Severe pre-mortem cardiac conduction abnormalities in Hutchinson-Gilford progeria syndrome (HGPS) minipigs. **a** An implantable loop recorder used for continuous electrocardiography (ECG) monitoring after subcutaneous implantation in wild-type (WT) and HGPS minipigs. **b** Representative single-lead ECG trace from a WT minipig. **c** ECG trace from a HGPS minipig, showing a wide QRS complex and short-duration polymorphic ventricular tachycardia before complete atrio-ventricular block at the moment of death. **d** ECG trace from a HGPS minipig, showing ST-segment elevation immediately before complete atrio-ventricular block at the moment of death. Red arrows in **b**–**d** indicate the P waves on the surface ECG; blue asterisks indicate the QRS complex. Traces correspond to a standard sweep speed of 25 mm/s and standard amplitude calibration of 10 mm/mV

Cardiac electrical activity was monitored continuously in vivo by placing implanted loop recorders (ILR) subcutaneously in five WT and four HGPS minipigs (Fig. 6a). Single-lead ECG recordings were obtained at the moment of death in three out of four HGPS minipigs. The WT minipigs did not show abnormal single-lead ECG parameters in the ILR (Fig. 6b). In contrast, three HGPS minipigs showed an advanced third degree atrio-ventricular block (total interruption of atrio-ventricular conduction) at the moment of death (Fig. 6c, d). In one HGPS minipig, complete atrio-ventricular block was preceded by a short-duration polymorphic ventricular tachycardia (Fig. 6c). Moreover, in another HGPS minipig the atrio-ventricular block was immediately preceded by ST-segment elevation, which was not present 29 h before death (Fig. 6d). This observation suggests a coronary ischemic event potentially related to microvascular dysfunction and vascular fibrosis (cf. Fig. 3).

### Other alterations in HGPS minipigs

MRI analysis of brain showed no cerebrovascular disease in the HGPS minipigs. None of the images indicated any localization with hyper-intense regions on FLAIR images or hypo-intense regions on T1-weighted images, which would be indicative of localized regions of stroke. Vascular microbleeds were not detected, as indicated by the lack of localized signal decay in T2*-weighted images (data not shown).

Gross histological examination of kidney, testicle, spleen, liver, and lung revealed pathological features only in the kidneys and testicles of HGPS minipigs. In the case of kidney, 88% of the HGPS minipigs showed dilation of cortical tubules and different degrees of glomerulosclerosis (Supplementary Fig. S6a). Testicles in the HGPS minipigs showed signs of delayed maturation, including a low number of interstitial Leydig cells and a near absence of mature spermatids (Supplementary Fig. S6b).

It proved impossible to establish an HGPS minipig colony through conventional breeding because disease symptoms in the male HGPS minipigs became severe during the period corresponding to pubertal development (from ~4 to 6 months of age) and none of the pigs survived to a post-pubertal age. As an alternative strategy, we collected and froze epididymal sperm from three necropsied HGPS minipig testicles and attempted surgical intrauterine insemination of two gilts treated with hormones to induce ovulation. One gilt received 300 × 10^6^ spermatozoids with 78.6% viability, 45% motility, and 62% acrosomal damage, and the other received 87.5 × 10^6^ spermatozoids with 46.4% viability, 15% motility, and 36% acrosomal damage. Ultrasonography revealed an absence of pregnancy 28 days after insemination in both gilts.

## Discussion

The generation of a large animal model for HGPS addresses the need for a more translational preclinical model of this devastating disease. Like patients carrying the heterozygous *LMNA* c.1824C > T mutation, the HGPS minipigs co-express endogenous progerin, lamin A and C, thus circumventing the limitation of HGPS-like mouse models lacking or overexpressing A-type lamin isoforms^2,11^. Progerin expression in the HGPS minipigs induced all major progeroid symptoms that, like in patients, are variable in severity along the time-course of disease, increase with age and culminate in early death. We focused our studies on CVD, which is considered the main cause of death in HGPS.

LV diastolic dysfunction was the most frequent echocardiographic abnormality in a cohort of 27 HGPS patients, with prevalence higher in the oldest patients^7^. LV systolic dysfunction and valve disease were infrequently seen in the first decade of life but became more prevalent in the second decade^7^. HGPS minipigs develop both systolic and diastolic dysfunction (including left atrial enlargement suggesting elevated LV filling pressure), which are common underlying causes of heart failure. Also consistent with reports in HGPS patients^6,7,32,37–40^, we found signs of mild mitral valve degeneration accompanied by mild mitral regurgitation in two of eight HGPS minipigs.

Prakash et al. reported normal ECG parameters in 25 of 27 HGPS patients^7^. However, ECG alterations were more prevalent (47%) in our previous study of 15 HGPS patients and 13 sex- and aged-matched healthy volunteers^8^. This apparent discrepancy may reflect differences in the median age at evaluation in the two cohorts (median age of 5.6 [2–17] years^7^ versus 11.5 [2–19] years^8^), with the older cohort predicted to have a greater burden of ECG alterations^9^. Notably, the HGPS minipigs exhibited significant ECG abnormalities at advanced disease stages, in particular longer QRS complex duration and significant bradycardia, in agreement with ECG alterations reported in progeroid mice at advanced disease stages^8^. The HGPS minipigs also showed Cnx43 mislocalization in the heart as an underlying cellular alteration related to ECG abnormalities, consistent with previous findings in HGPS patients and progeroid mice^8^. We further investigated cardiac electrical activity through continuous in vivo recordings, which revealed pre-mortem advanced atrio-ventricular conduction block in three out of four HGPS minipigs. Moreover, the short-duration polymorphic ventricular tachycardia observed in one HGPS minipig before complete atrio-ventricular block (likely related to severe bradycardia) is in agreement with our previous findings demonstrating premature ventricular complexes in progeroid mice during sequential ECG examination^8^. Another HGPS minipig had overt ST elevation at the moment of death, suggesting that myocardial ischemia may have contributed to the atrio-ventricular block. Consistent with this, our in vivo cardiac MRI studies identified reduced myocardial perfusion and interstitial fibrosis, both contributing to the diastolic LV dysfunction phenotype in the HGPS minipigs. These MRI findings are confirmed by the histopathological studies revealing a significant proportion of degenerated medium-to-large coronaries and above-normal collagen deposition in cardiac arterioles and myocardial tissue. The defective myocardial perfusion and interstitial fibrosis are previously unidentified cardiovascular alterations in genetically diagnosed HGPS patients that deserve attention when monitoring disease progression in patients and can be used as in vivo readouts in clinical trials. Our HGPS minipig model can guide future research into the natural history of CVD in HGPS and aid in the important task of elucidating primary molecular and cellular mechanisms of progerin-induced damage. These knockin minipigs should also help to identify early biomarkers of disease progression and responses to therapies.

The course of disease in the HGPS minipigs correlates well with the timing of disease onset, progression, and death in patients, with both species manifesting symptoms a certain period after birth and dying around puberty. With a mean life span of just ∼6 months, our HGPS minipig model is ideal for preclinical studies, because it offers a wide window for therapeutic interventions to prevent the appearance of symptoms or revert the phenotype, while also permitting longevity studies over a relatively short period. This favorable timing will enormously facilitate the execution of future preclinical tests. Indeed, this HGPS minipig model can provide an appropriate filter for drug-based strategies with proven efficiency in HGPS-like mice^12^ before moving them forward to clinical trials. The expectation is that any preclinical tests in HGPS minipigs will yield highly translatable results regarding drug regimes, administration routes, bioavailability, pharmacokinetics, efficacy, and acute toxicity. Likewise, these knockin minipigs should help in the development and optimization of cell-based therapies and systemic or tissue-specific gene therapy. Notably, the c.1824C > T mutation in the pig *LMNA* gene provokes progerin expression, suggesting that HGPS minipigs may be useful for testing therapies targeting aberrant *LMNA* exon 11–12 splicing, such as morpholino-oligonucleotides, which showed efficacy in progeroid mice^33^ but await testing in a large animal model.

The human-like size of the HGPS minipig model is another key advantage that allowed the use of the same MRI, echography, ECG, and telemetry devices used in hospitals for human diagnosis. Due to dentition and mandibular problems, anesthesia is considered a risky procedure in HGPS patients. The HGPS minipigs recapitulate these dental and facial bone alterations, and due to the narrow airway in these animals, standard intubation for anesthesia was substituted in some cases by ventilation with canine laryngeal masks. Any interventional trial in HGPS minipigs involving anesthesia will therefore face similar challenges to those encountered with HGPS patients, and will thus produce invaluable translational results. For all these reasons, we expect this progeroid minipig model to have a strong and positive impact on HGPS by expediting the development of effective therapies for HGPS patients.

The difficulty encountered in establishing a HGPS minipig colony either by mating HGPS boars with WT sows or by surgery-assisted fertilization of sows with sperm obtained from HGPS minipigs was unsurprising given the lack of fitness and delayed pubertal development among the male HGPS minipigs. Although we were able to cryopreserve and freeze small amounts of sperm from HGPS necropsied testicles, attempts to surgically inseminate two hormone-treated gilts were unsuccessful. Therefore, the generation of new HGPS minipigs for future preclinical trials will require either in vitro fertilization or recloning using our heterozygous *LMNA* 1824C > T Yucatan minipig fibroblast clone or fibroblasts isolated from HGPS minipigs. While disadvantageous from the perspective of colony generation, the lack of reproductive fitness is a further example of the recapitulation of disease symptoms seen in patients, who experience delayed or absent pubertal development. For example, a 12-year-old HGPS female patient and a 17-year-old HGPS male patient were classified as Tanner developmental stage II (first appearance of pubic hair and breast buds, and a slight enlargement of penis and testicles, respectively)^6^. Additionally, a group of female HGPS patients aged over 12 years and who declared having experienced menarche remained between the Tanner I and II pubertal stages even 2 years after their menarche^41^. To our knowledge, there are no reports of the potential viability of gametes from HGPS patients. Although the dose of A-type lamins can affect spermatogenesis in rodents^42^, A-type lamins were not detected in human spermatids or mature spermetazoa^43^. Consequently, no progerin-induced damage would be expected in mature HGPS spermatozoids. Recently, it was reported that mouse *Lmna*^*G609G/G609G*^ oocytes are meiotically competent and can be fertilized by WT spermatozoids and that *Lmna*^*G609G/G609G*^ sperm, although not abundant, is motile and able to fertilize WT oocytes^44^. The same authors propose that the sub-fertility in *Lmna*^*G609G/G609G*^ mice may simply be a consequence of the lack of physical fitness. This idea is supported by the physical condition of the HGPS minipigs and the low numbers of interstitial Leydig cells and mature spermatids, indicating delayed pubertal development. These findings may reflect a failure of the minipigs to achieve the necessary weight or fat threshold to trigger sexual maturation.

A potential limitation of our study is that we only included male HGPS minipigs because all the cloned animals were derived from genetically modified fibroblasts obtained from a male Yucatan minipig. However, no gender-specific alterations have been reported in HGPS patients, and we expect future preclinical results generated in our HGPS minipig model to be equally translatable to male and female patients.

We believe that our heterozygous *LMNA* c.1824C > T Yucatan minipig model is a bona fide and necessary bridge between HPGS mouse models and patients. These knockin minipigs provide an appropriate large animal model in which to test human-size interventional devices (like stents or pacemakers), and standardize surgical models or medical protocols using the same methodologies utilized with patients. Importantly, HGPS minipigs will facilitate the optimization of drug regimens escalated from small- to large-body-size mammals, and will accelerate the unraveling of potential counteracting toxicities in combined new therapies, or side effects in challenging future cures. This model will therefore facilitate decision-making about which therapeutic strategies should advance to clinical trials and potentially expedite the development of effective therapeutic applications for HGPS patients. Moreover, as progerin is expressed at low levels in tissues from aged non-HGPS individuals (reviewed in ref. ^2^), we believe that research in this HGPS minipig model may also shed light into the complex mechanisms underlying physiological aging.

## Materials and methods

The data, analytic methods, and study materials will be made available to other researchers for purposes of reproducing the results or replicating the procedure (available at the authors’ laboratories). Supplementary Fig. S2a shows an overview of the method for generating the HGPS minipigs. Detailed protocols of all the steps followed for generating HGPS minipigs and assisted reproduction, and expression studies are in Supplementary Information.

### Study approval

All animal procedures at Aarhus University (AU) were approved by the Danish Animal Experiments Inspectorate (licence no. 2014-15-0201-00434). Experimental procedures at the Centro Nacional de Investigaciones Cardiovasculares (CNIC), Universidad de Murcia, and the Spanish National Institute for Agricultural and Food Research and Technology conform to EU Directive 2010/63EU and Recommendation 2007/526/EC, enforced in Spanish law under Real Decreto 53/2013. Animal protocols were approved by the respective local ethics committees. Animal work at the CNIC was approved by the Comunidad Autónoma de Madrid (PROEX #313/16). The cloned HGPS animals were generated at AU and housed in a specific pathogen-free facility under standard conditions. At ~3.5 months of age, animals were transported to Agropardal S.L. (Cuenca, Spain), a farm dedicated exclusively to the breeding of experimental pigs. Phenotypic characterization of minipigs was conducted at the CNIC’s animal facility. Surgical intrauterine insemination of gilts was carried out at the Universidad de Murcia. All minipigs were uncastrated Yucatan males except for two WT minipigs that were castrated and excluded in weight and survival studies. We used non-littermate WT controls because it was not possible to cross the male HGPS minipigs with WT gilts.

### Anesthesia

Anesthesia was administered to all Yucatan minipigs before proceeding with non-invasive procedures and implantation of a telemetry monitor. Anesthesia was induced by intramuscular injection of ketamine (20 mg) followed by continuous intravenous infusion of ketamine (2 mg/kg/h), xylazine (0.2 mg/kg/h), and midazolam (0.2 mg/kg/h) throughout the protocol.

### Echocardiography

Diastolic function patterns were studied with a Commercial scanner (iE33, Philips Healthcare) and the standard techniques^45,46^. Likewise, valve function was analyzed according to European Society of Cardiology guidelines^45^. Ultrasound images were analyzed with Qlab R 9.1 (Philips Medical Systems) by two expert observers blinded to genotype. Pulsed-wave Doppler imaging was used to determine mitral valve inflow velocity during early (*E*) and late (*A*) diastole, *E*/*A* velocity ratio, mitral *E* velocity DT, septal early (*E*′) diastolic myocardial velocity, and *E*/*E*′ ratio. In addition, left atrium antero-posterior dimensions were obtained and normalized to body surface area using the modified Brody’s formula^47,48^. Images were analyzed by two researchers blinded to genotype.

### Cardiac MRI

CMRI studies were performed in a 3 Tesla Philips Achieva Tx whole-body scanner (Philips Healthcare) equipped with a 32-element phased-array cardiac coil. The imaging protocol included anatomical and functional assessment using a segmented cine steady-state free-precession (SSFP) sequence with spatial resolution of 1.8 × 1.8 mm, end-diastolic acquisition, thickness 6 mm with no gap, and 30 fully acquired cardiac phases. Segmented cine SSFP was performed to acquire 11–13 contiguous short-axis slices covering the heart from the base to the apex in order to evaluate global and regional LV motion. Multiparametric tissue characterization was performed using T2 mapping and pre- (native) and post-contrast T1 mapping techniques and absolute quantitative perfusion. T2 mapping data were acquired using a previously validated T2-GraSE sequence with eight equally spaced echo-times ranging from 6.7 to 53.7 ms^49^. T1 mapping images were captured using a 5(3)3 MOLLI scheme with a single-shot balanced acquisition (TR/TE/Flip, 2.1 ms/1.05 ms/35°) and a spatial resolution of 1.5 × 1.8 × 8.0 mm^3^. Tissue characterization parameters were acquired from a single mid-ventricular slice in short-axis orientation. Microvascular status was studied with a previously reported methodology to assess absolute quantitative perfusion using a dual saturation recovery sequence^30^. Macro areas of increased extracellular space (a fibrosis surrogate) were assessed by delayed gadolinium enhancement (10–15 min after intravenous administration of 0.20 mmol gadobutrol contrast agent per kg body weight) using a T1-weighted three-dimensional inversion recovery sequence with an adjusted inversion time to null the healthy myocardium and an isotropic resolution of 1.5 × 1.5 × 1.5 mm^3^. CMR images were analyzed using dedicated software (MR Extended Work Space 2.6, Philips Healthcare, and QMass MR 7.6, Medis) by two observers experienced in CMR analysis who were blinded to genotype. LVEF, LV volumes, and LV mass were calculated by tracing endocardial/epicardial borders during end-systole and end-diastole in short-axis slices. Mapping analyses were performed with a region of interest in a mid-apical LV short-axis slice. All volumes and masses were normalized to body surface area using the modified Brody’s formula^47,48^. Images were analyzed by two researchers blinded to genotype.

### Electrocardiography

The 12-lead ECG recordings were obtained before imaging studies using the same anesthesia period. ECG recordings were acquired at 1 kHz for at least 5 min (LabSystem-Pro recording system, Boston Scientific). PR, QRS, and QT intervals were measured using manual calipers at a 200 mm/s sweep speed. QRS complex duration was measured as the time from the lead showing the earliest deflection (positive or negative) to the lead with the latest offset of the QRS complex^50^. QT intervals were corrected with the Bazzet formula. A set of 10 beats per animal was analyzed and the mean value of each ECG parameter was assigned to the animal. Two researchers blinded to the genotype performed the analysis.

### Implantable loop recorder placement

ILR (Reveal Linq, Medtronic) were placed subcutaneously in four HGPS and five WT Yucatan minipigs under local anesthesia with lidocaine 1% (5 ml, 1 mg/ml vials). The device was placed subcutaneously on the left side of the sternum to ensure accurate QRS complex recordings.

### Hematology and metabolic profile

Blood samples were analyzed in an ADVIA 2120i hematology system (Siemens). Serum samples were analyzed in a Dimension RxL Max analyzer (Siemens).

### Statistics

Statistical significance was assessed by unpaired two-tailed Student’s *t*-test using GraphPad Prism software, except for the % fibrosis in LV and septal myocardium in Fig. 3e, for which an unpaired one-tailed Student’s *t*-test was used. A log-rank (Mantel-Cox) test was used for the Kaplan-Meier survival curve. Differences were considered significant at *p* < 0.05.

## Supplementary information

Supplementary Information

Supplementary Movie S1

Supplementary Movie S2

Supplementary Movie S3

Supplementary Movie S4

Supplementary Movie S5

## Supplementary information

**Supplementary Information** accompanies the paper at (10.1038/s41421-019-0084-z).

## Note

After acceptance of this article, the authors have generated new HGPS minipigs by SCNT using heterozygous *LMNA* 1824 C>T Yucatan minipig fibroblasts (in collaboration with Dr. Eckhard Wolf, Ludwig-Maximilians-Universität München, Germany).
