# Supplementary material for: Generation and characterization of a novel knockin minipig model of Hutchinson-Gilford progeria syndrome
Source: Cell Discov. 2019 Mar 19;5:16. doi: 10.1038/s41421-019-0084-z (PMC6423020; doi:10.1038/s41421-019-0084-z)
Supplement: Supplementary file 1 — Supplementary Information [file 41421_2019_84_MOESM1_ESM.pdf]

CONTENT

- **Supplementary Methods.**
- **Supplementary Fig. S1: The "classical" HGPS silent *LMNA* c.1824C>T mutation promotes progerin expression.**
- **Supplementary Fig. S2: Generation of knockin HGPS Yucatan minipigs.**
- **Supplementary Fig. S3: Progeroid external features in HGPS minipigs.**
- **Supplementary Fig. S4: Microvascular dysfunction in the hearts of HGPS minipigs.**
- **Supplementary Fig. S5: Unaffected systolic and diastolic parameters in HGPS minipigs.**
- **Supplementary Fig. S6: HGPS minipigs develop glomerulosclerosis and delayed testicular maturation.**
- **Supplementary Table S1: Molecular tools for CRISPR-Cas9-mediated gene editing of *LMNA* exon 11 in Yucatan minipig skin fibroblasts.**
- **Supplementary Table S2: Nucleotide sequences of primers used for molecular assays.**
- **Supplementary Table S3: Analysis of serum from wild-type and HGPS minipigs.**
- **Supplementary Movies S1-S5.**

Cell culture

Primary porcine fibroblasts were established by explant culture of ear biopsies obtained from newborn male Yucatan minipigs. Until fibroblast outgrowth, ear biopsies were cultured in AmnioMAX-C100 complete medium (Thermo Fischer Scientific, cat. no. 12558011). Following isolation, fibroblasts were cultured at 37°C in Dulbecco´s Modified Eagles Medium (DMEM, Lonza Biowhittaker, cat. no. BE12-604F) supplemented with 15% heat-inactivated fetal calf serum, 60 µg/mL penicillin, 100 µg/mL streptomycin, and 292 µg/mL glutamine. For gene editing experiments, the cell culture medium was also supplemented with basic fibroblast growth factor (bFGF, 5 ng/mL).

CRISPR/Cas9 pLMNA sgRNAs

The human codon-optimized Cas9 (gift from George Church, Addgene plasmid #41815) and sgRNAs were expressed from separate plasmids. CRISPR/Cas9 sgRNAs targeting the porcine *LMNA* gene were designed using the online sgRNA design tool Zifit (https://crispr-cas9.com/96/zifit-targeter-crispr-cas9/). Three individual sgRNAs were designed to span the p*LMNA* region comprising the targeted nucleotide, cloned into plasmids, and validated before gene editing of porcine fibroblasts[1]. For each sgRNA construct, two p*LMNA*-specific complementary oligonucleotides were denatured and slowly annealed before ligation of the annealed oligonucleotides to a sgRNA scaffold plasmid (pFUS-U6-sgRNA) based on *Bsa*I assembly[1]. The ligation mixture was used to transform XL-2 Blue ultracompetent bacterial cells (Agilent Technologies, cat. no. 200150). The resulting bacterial cell clones were screened by PCR, and positive sgRNA clones were validated by DNA sequencing of isolated plasmid DNA. Oligonucleotides and target sites used for generation of the sgRNA vectors are listed in **Supplemental Table S1.**

pLMNA-specific C-check vector

The three generated sgRNAs were validated using a previously developed single strand annealing (SSA)-directed, dual fluorescent surrogate reporter system called C-check. This vector comprises two expression cassettes: a truncated *EGFP* expression cassette for detection of double strand break (DSB)-induced SSA events and an *AsRED* expression cassette for measuring transfection efficiency and for normalization[1]. The *EGFP* cassette is interrupted by one or a series of target sites for the sgRNAs of interest. Upon sgRNA-induced DSB in this target region, the C-check reporter construct will express *AsRED* and *EGFP* if repaired by SSA, whereas only *AsRED* will be expressed if no DSB is induced or repair occurs by non-homologous end joining. The p*LMNA*-specific C-check vector was constructed as described[1]. Briefly, two complementary oligonucleotides comprising the sgRNA target site(s) were annealed and cloned into the *Bsa*I-digested C-check vector. The ligated plasmid was used for bacterial cloning in XL-2 Blue Ultracompetent cells[1]. The resulting bacterial cell clones were PCR screened, and positive C-check clones were validated by DNA sequencing of isolated plasmid DNA. The oligonucleotides used to generate the pLMNA-specific C-check vector comprising overlapping sgRNA1, sgRNA2, and sgRNA3 target sites are listed in **Supplementary Table S1**.

Flow cytometry evaluation of pLMNA gRNA efficiency

The efficiency of the three generated sgRNAs was evaluated by transfection into HEK293T cells and flow cytometry of the transfected cells[1]. The cells were seeded the day before transfection into 6-well plates (3 x10^5 cells/well). The next day, cells were co-transfected with one of the three sgRNAs plasmids (75, 150, and 300 ng) together with the hCas9 plasmid (75, 150, and 300 ng), the p*LMNA* specific C-check plasmid

(300 ng), and stuffer plasmid DNA (to adjust the amount of total DNA to 1 µg) using X-tremeGENE 9 DNA transfection reagent (Sigma-Aldrich). As controls, cells were transfected with only the C-check or hCas9 plasmid, or with sgRNA plasmid together with the C-check vector. At 48 hours post transfection, the cells were harvested by trypsinization and analyzed by flow cytometry in a BD FACSARIA III sorter (BD Bioscience) (FACS Core Facility, Dept. of Biomedicine, AU) to quantify the efficiency (*EGFP* expression) of the individual sgRNAs in transfected (*AsRED*$^+$) cells.

pLMNA c.1824C>T donor plasmid

A donor plasmid comprising the homologous p*LMNA* sequence including the c.1824C>T mutation was constructed by 2-fusion PCR. Two PCRs with overlapping sequences were run, each using a Platinum Pfx DNA polymerase, genomic DNA isolated from a WT male Yucatan minipig as template DNA, and primer sets including a primer containing the C>T mutation. The two resulting overlapping c.1824C>T-containing amplicons were used as a template (100 ng of each amplicon per 25 µl PCR reaction) in a 2-fusion PCR resulting in a 378-bp product comprising a 236-bp left homology arm (LHA) and a 141-bp right homology arm (RHA) flanking the p*LMNA* C>T mutation. The HGPS-causing *LMNA* c.1824C>T mutation results in aberrant splicing of exon 11 and exon 12. To mimic this, and to avoid eventual additional interference with the intended splicing of the gene-edited c.1824C>T p*LMNA* allele, the PAM site located downstream of the sgRNA1 recognition site in the donor p*LMNA* sequence was left intact. The 2-fusion PCR was performed using Platinum Pfx polymerase and the following PCR protocol: 94°C/2´; 35x (94°C/20´´- 62°C/30´´- 68°C/30´´), 68°C/7 min. Fusion products were cloned using a Zero Blunt TOPO PCR cloning kit (Invitrogen, cat. no. 450245) and the ligation mixture used for transformation of XL-2 Blue Ultracompetent cells. The resulting bacterial colonies were PCR screened before plasmid DNA isolation from positive bacterial cell clones. Finally, the p*LMNA* c.1824C>T plasmid was verified by DNA sequencing before being used as a donor plasmid for gene editing of porcine fibroblasts. Primers used to generate the pLMNA c.1824C>T plasmid are listed in **Supplementary Table S1.**

Transfection of primary porcine fibroblasts and expansion of gene-edited cells

Primary fibroblasts from newborn male Yucatan piglets were seeded onto a gelatin-coated 10 cm cell culture dish one day before transfection (1.5×10^6 cells). Before transfection, the culture medium was changed and supplemented with bFGF (5 ng/µL). The cells were then co-transfected with the sgRNA1 vector (500 ng), hCas9 vector (1500 ng), the p*LMNA* c.1824C>T donor construct (2500 ng), and EGFP-N3 plasmid (1000 ng, Clontech) using Lipofectamine LTX with Plus Reagent (Thermo Fischer Scientific, cat. no. 15338100). The transfected cells were harvested by trypsinization 48 hours post transfection and analyzed by FACS (FACS Core Facility, Dept. of Biomedicine, AU) to enrich for gene-edited cells based on EGFP expression. Propidium-iodide-negative EGFP-expressing cells were sorted using a BD FACSARIA III sorter and were seeded in 7 gelatin-coated 96-well plates (3 cells per well). Single cell clones were subsequently expanded by culturing in 15% DMEM supplemented with 5 ng/ml bFGF. At subconfluency, the cell clones were trypsinized, and 1/2 of the resulting cell suspension was transferred to 96-well PCR plates for lysis and PCR screening. The remaining half of the cell suspension was cultured in gelatin-coated 96-well cell culture plates for further expansion and freezing at early passages of PCR-validated gene-edited cell clones before their use as nuclear donor cells for SCNT.

PCR screening and DNA sequencing of gene edited donor cells

EGFP$^+$ cells in 96-well PCR plates were centrifuged and re-suspended in 25 µL lysis buffer (50 mM KCl, 1.5 mM $MgCl_2$, 10 mM Tris-Cl, pH 8.5, 0.5% Nonidet P40, 0.5% Tween, 400 µg/ml Proteinase K)[2]. The cells were lysed (65°C for 30 mins) followed by proteinase K inactivation (95°C for 10 mins), and 1 µL

lysate was used as template for allele-specific PCR screening. A forward primer (5´-GCCTCTCAAGCCCTGTCACC-3´) and an allele-specific reverse primer (5´-GAGCCAGAGGAGATGGATCCA-3´) discriminating between the WT and gene-edited pLMNA alleles were used with the following PCR conditions: 94°C/3´, 35x (94°C/30´´- 70°C/30´´- 72°C/20´´), 72°C/7´. Negative controls for the PCR screen were WT Yucatan minipig genomic DNA and water. Cell clones found to be positive for the *pLMNA* c.1824C>T mutation by the PCR described above were further analyzed by two PCR protocols to validate the fidelity of the 5´- and 3´-gene-targeting events. These PCR protocols used Platinum Pfx DNA polymerase and primer sets consisting of primers located outside and internally in the targeted region, respectively **(Supplementary Table S2).** Successfully gene-edited cell clones (positive for all three PCRs) were further verified by DNA sequencing of the amplicons resulting from both the 5´- and the 3´-gene-targeting PCRs prior to being selected as donor cells for hand-made cloning.

Off-target analysis of gene edited cell clones

The CRISPR RGEN Cas-OFFinder algorithm was used to identify potential off-target sites for the employed sgRNA1. Allowing for up to 3 mismatches between the sgRNA and genomic sequence, 9 genomic regions, in addition to the targeted *pLMNA* target gene, were identified on chromosomes 1, 5, 6, 8, 11, and 13. Two potential off-target sites reside in annotated genes on chromosome 6 (*USB1* and *PRKCZ*). Except for one locus on chromosome 5, which proved difficult to amplify, all potential off-target regions were amplified by standard PCR using genomic DNA from WT or gene-edited Yucatan cells clones and a Platinum Pfx DNA polymerase. The resulting amplicons were subjected to DNA sequencing to verify if off-target activity had occurred.

In addition, the gene-edited cell clones were analyzed for potential unwanted random integration of the plasmid constructs used for co-transfection (sgRNA, hCas9, EGFP-N3, and the donor plasmid). These analyses were performed by standard PCR using genomic DNA from either WT or gene-edited cells and primer sets specific for the individual plasmids used for transfection. Primers used for off-target and random integration analyses are shown in **Supplementary Table S2.**

Genotyping of cloned gene edited piglets

Genotyping of the cloned *pLMNA* c.1824C>T gene-edited minipigs was performed by standard PCR using the allele-specific PCR and the 3´- gene-targeting PCRs mentioned above **(for primer sequences, see Supplementary Table S2)**. Gene editing of the cloned piglets was also verified by DNA sequencing of amplicons resulting from a 5´+ 3´-gene targeting PCR essentially combining the 5´- and 3´-gene targeting PCRs but using Platinum Pfx DNA polymerase and primers flanking the entire targeted DNA region **(for primer sequences, see Supplementary Table S2).**

Handmade cloning, embryo culture and transfer, pregnancy period, and delivery of piglets

Handmade cloning was performed as described[3]. Cumulus-oocyte complexes harvested from slaughterhouse-derived ovaries were in-vitro matured and treated to remove cumulus cells and partially remove zonae pellucidae. The oocytes were bisected manually, and the chromatin-free cytoplasts were collected. Each cytoplast was first attached to one knockin HGPS fibroblast cell before fusion (BTX microslide 0.5 mm fusion chamber, model 450; BTX San Diego, US). After 1 h incubation, each cytoplast-fibroblast pair was fused with an additional cytoplast to create the reconstructed embryo. All reconstructed embryos were then incubated in culture medium for 5-6 days, when blastocysts and morulae were selected based on morphology. Two pools of cloned transgenic embryos were prepared, and respectively 92, 66 and 70 blastocysts/morulae were transferred surgically into three recipient landrace surrogate sows[4]. Pregnancy was diagnosed in all sows by ultrasonography after approximately 25 days. Farrowing was hormonally initiated at day 114 by

intra-muscular prostaglandin injection. The sows farrowed a total of 19 piglets (15, 3 and 1), of which 10 were still alive after two weeks **(Supplementary Fig. S2b).**

Epididymal sperm extraction

Immediately after death, the testes with attached epididymes and vas deferens of 3 HGPS boars were placed in a portable cooler at 18-20ºC and transported for processing to the Physiology and Biotechnology of Reproduction in Swine Laboratory at the INIA. Epididymal samples were collected following a previously reported flushing technique[5]. The cauda epididymis and ductus deferens were isolated from the rest of the epididymis by making a cut near the junction of the corpus and the proximal cauda. For epididimal fluid collection, the vas deferens cannulated with a blunted 21G needle, and epididimal fluid was collected by retrograde washing from the vas deferens and epididymis tail using a syringe loaded with 1 mL Beltsville thawing solution (BTS) extender. The vas deferens and the cauda region were perfused with the extender, followed by air injection, until all the contents were removed from the cauda epididymis. Samples from each testicle were collected in 10 ml sterile plastic tubes and cryopreserved for storage in liquid nitrogen.

Surgical intrauterine insemination of wild-type gilts

Two gilts (approximate 5.5 months old and weighing 90-95 kg) were induced to ovulate with sequenced injection of eCG (1500 IU, Foligon; Intervet International BV, Boxmeer, Holland) followed by hCG (750 IU Veterin Corion; Divasa Farmavic, Barcelona, Spain) 72 h after the first injection[6]. The expected time of ovulation was 38-42 h after hCG injection[7]. Pigs were kept in individual pens, and after a 24 h fast, they were pre-medicated with an intramuscular injection of morphine (0.2 mg/kg), metadomidine (0.2 mg/kg), and ketamine (10 mg/kg). Anesthesia was maintained with isofluorane (ISOFLO, Esteve 2-3% O_2)[8]. Animals were placed in lateral recumbency and under CO_2 pneumoperitoneum of 8-10 mmHg. Paralumbar laparo-endoscopic single-site surgery (LESS) was carried out using a monoport device (GelPOINT Advanced Access Platform, Applied Medical, Rancho Santa Margarita, California, USA). After rapid visualization of the abdominal organs, non-traumatic laparoscopy forceps were used to grasp the ad-ovarian end of the uterine horn towards them on port opening. Pneumoperitoneum and the monoport cap were then removed to allow gentle manipulation of the reproductive organs[9]. Frozen semen samples were thawed at 37º for 30 seconds and diluted in tempered BTS[10]. Semen doses were deposited into the oviduct with a blunt needle[11]. The gilts were kept under the usual farm conditions, and pregnancy status was assessed by ultrasonography 25-28 days after insemination (100 FALCO-VET scan, Esaote Espana S.A., Barcelona, Spain).

Progerin expression

Total mRNA was extracted in Tri-Reagent solution (Invitrogen AM9738), quantified in a NanoDrop spectrophotometer (Wilmington), and retrotranscribed to cDNA using the High Capacity cDNA Reverse Transcription Kit (Applied Biosystems). PCR to detect progerin mRNA was performed with a forward primer (5´-GCAACAAGTCCAATGAGGACCA-3´), a reverse primer (5´-CATGATGCTGCAGTTCTGGGGGCTCTGGAC-3´), 100-200 ng of cDNA, and 1 U/ml of Taq DNA polymerase (Biotools) in a c1000 Thermal Cycler (Bio-Rad). The 482-bp PCR product was purified after agarose gel separation and subjected to DNA sequencing. The sequence was analyzed with Chromas software.

Protein extracts were obtained in lysis buffer (50 mM Tris HCl pH 7.5, 150 mM NaCl, 0.1% SDS, 0.5% sodium deoxycholate, 1% NP-40, 1 mM DTT) containing protease and phosphatase inhibitors (Roche) by mechanical disruption with POLYTRON® PT 6100 homogenizers (Kinematica) and sonication with a Bioruptor (Diagenode) at 4ºC. Protein extracts (30-60 μg) were separated on 10% SDS-PAGE gels and

transferred to PVDF membranes (Immobilon-P, Merck Millipore). Primary antibodies used for immunoblot were anti-lamin A/C (Santa Cruz Biotechnologies sc-376248) and anti-glyceraldehyde-3-phosphate dehydrogenase (anti-GAPDH, Merk Millipore MAB374), and the secondary antibody was m-IgGκ BP-HRP (Santa Cruz biotechnologies sc-516102). Blots were developed with Luminata Forte Western HRP Substrate (Millipore WBLUF0100), and images were acquired with ImageQuant LAS 4000 mini (GE Healthcare Lifesciences).

Histology and immunofluorescence

Tissues were fixed in 4% paraformaldehyde and embedded in paraffin. Serial 4 µm sections were stained with hematoxylin-eosin (H&E) and Masson trichrome (MT). Antigen retrieval was performed with 10 mM sodium citrate buffer (pH 6). Samples were blocked for 1 h at room temperature (RT) with 100 mM glycine and permeabilized for 1 h at RT in PBS containing 0.3% Triton X-100 (Sigma 9002-93-1), 5% normal goat serum (Jackson ImmunoResearch), and 5% bovine serum albumin (BSA, Sigma). Sections were then incubated overnight at 4°C with anti-CD31 (DIA-310 DIANOVA, 1:50), anti-N-cadherin (33-3900, ThermoScientific, 1:100) and anti-connexin 43 (Abnova PAB12759, 1:100). After washing, samples were incubated with corresponding secondary antibodies (goat anti-rabbit Alexa Fluor 647, Invitrogen A21245), a nucleic acid stain Hoechst 33342 (Sigma B2261) and anti-α-smooth muscle actin-Cy3 (Sigma SMA-Cy3 C6198) for 2 h at RT and then mounted in Fluoromount G imaging medium (Affymetrix eBioscience). Images were acquired with LSM 700 and 880 Carl Zeiss confocal microscopes. Stained sections were scanned with a NanoZoomer-RS scanner (Hamamatsu), and images were exported with NDP.view2 software and analyzed with ImageJ Fiji software by two researchers blinded to genotype. Collagen content in myocardial tissue was quantified by deconvolution of MT-stained images as the mean % of 7 areas distributed along the LV and 6 areas along the septum (470 μm^2 free of vessels). Coronary artery number and degeneration were analyzed in areas of 120 mm^2 in septum and 170 mm^2 in LV in MT-stained images of transverse heart sections. Connexin 43 and N-cadherin localization in heart tissue was quantified as the mean of three 236 μm^2 regions per minipig using ImageJ JACoP plugin[12].

REFERENCES SUPPLEMENTARY METHODS

1 Zhou, Y. *et al.* Enhanced genome editing in mammalian cells with a modified dual-fluorescent surrogate system. *Cell Mol Life Sci* **73**, 2543-2563 (2016).

2 McCreath, K. J. *et al.* Production of gene-targeted sheep by nuclear transfer from cultured somatic cells. *Nature* **405**, 1066-1069 (2000).

3 Li, J. *et al.* Developmental potential of pig embryos reconstructed by use of sow versus pre-pubertal gilt oocytes after somatic cell nuclear transfer. *Zygote* **22**, 356-365 (2014).

4 Schmidt, M. *et al.* Pregnancies and piglets from large white sow recipients after two transfer methods of cloned and transgenic embryos of different pig breeds. *Theriogenology* **74**, 1233-1240 (2010).

5 Martinez-Pastor, F. *et al.* Comparison of two methods for obtaining spermatozoa from the cauda epididymis of Iberian red deer. *Theriogenology* **65**, 471-485 (2006).

6 Krueger, C., Rath, D. & Johnson, L. A. Low dose insemination in synchronized gilts. *Theriogenology* **52**, 1363-1373 (1999).

7 Coy, P. *et al.* Environment and medium volume influence in vitro fertilisation of pig oocytes. *Zygote* **1**, 209-213 (1993).

8 Garcia-Vazquez, F. A. *et al.* Production of transgenic piglets using ICSI-sperm-mediated gene transfer in combination with recombinase RecA. *Reproduction* **140**, 259-272 (2010).

9 Garcia-Martinez, S. *et al.* Mimicking physiological O2 tension in the female reproductive tract improves assisted reproduction outcomes in pig. *Mol Hum Reprod* **24**, 260-270 (2018).

10 Selles, E., Gadea, J., Romar, R., Matas, C. & Ruiz, S. Analysis of in vitro fertilizing capacity to evaluate the freezing procedures of boar semen and to predict the subsequent fertility. *Reprod Domest Anim* **38**, 66-72 (2003).

11 Garcia-Vazquez, F. A. *et al.* Factors affecting porcine sperm mediated gene transfer. *Res Vet Sci* **91**, 446-453 (2011).

12 Bolte, S. & Cordelieres, F. P. A guided tour into subcellular colocalization analysis in light microscopy. *J Microsc* **224**, 213-232 (2006).

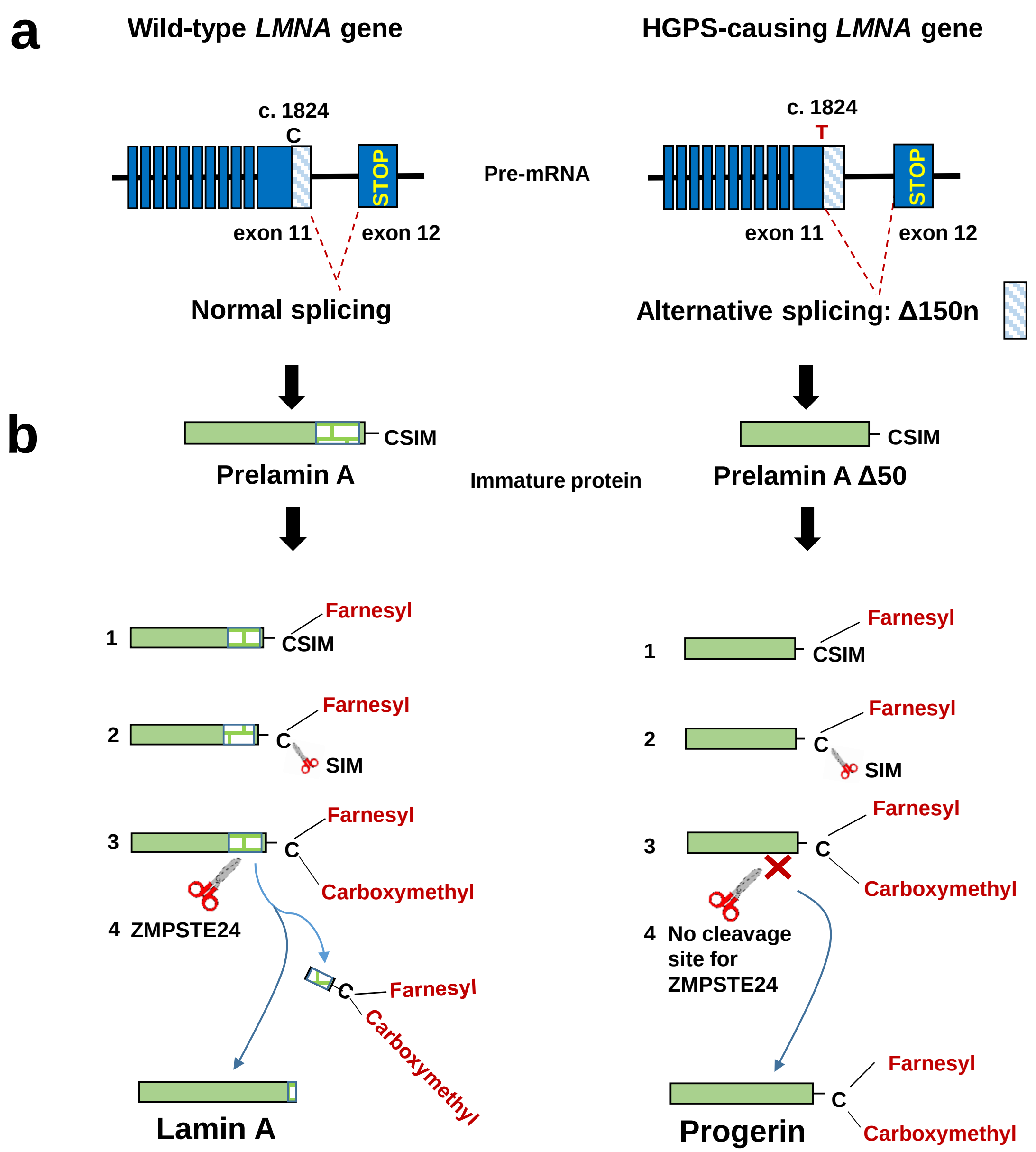

Supplementary Fig. S1. The "classical" HGPS silent *LMNA* c.1824C>T mutation promotes progerin expression.

(a) ***LEFT:*** WT *LMNA* gene pre-mRNA processing follows a normal splicing event between exons 11 and 12. ***RIGHT:*** The *LMNA* c.1824 C>T mutation promotes the use of an aberrant 5´ cryptic splice site in exon 11 that causes an in-frame deletion of 150 coding nucleotides.

(b) ***LEFT:*** The *LMNA* mRNA is translated into an immature form called Prelamin A. Posttranslational modifications of Prelamin A involve several sequential steps in the conserved CSIM residues in the C-terminus, including 1) farnesylation, 2) cleavage of the 3´-SIM residues by the protease ZMPSTE24, 3) carboxymethylation, and 4) cleavage by ZMPSTE24 to release the farnesylated and carboxymethylated C-terminal region and yield mature Lamin A. ***RIGHT:*** Due to aberrant splicing caused by the "classical" HGPS *LMNA* c.1824C>T mutation, mutant Prelamin A Δ50 lacks a 50 amino-acid sequence encompassing the ZMPSTE24 cleavage site. Therefore, the resulting protein, progerin, remains irreversibly farnesylated and carboxymethylated in its C-terminus. Progerin accumulates in the inner nuclear membrane and severely alters multiple cellular functions in a dominant-negative manner.

a

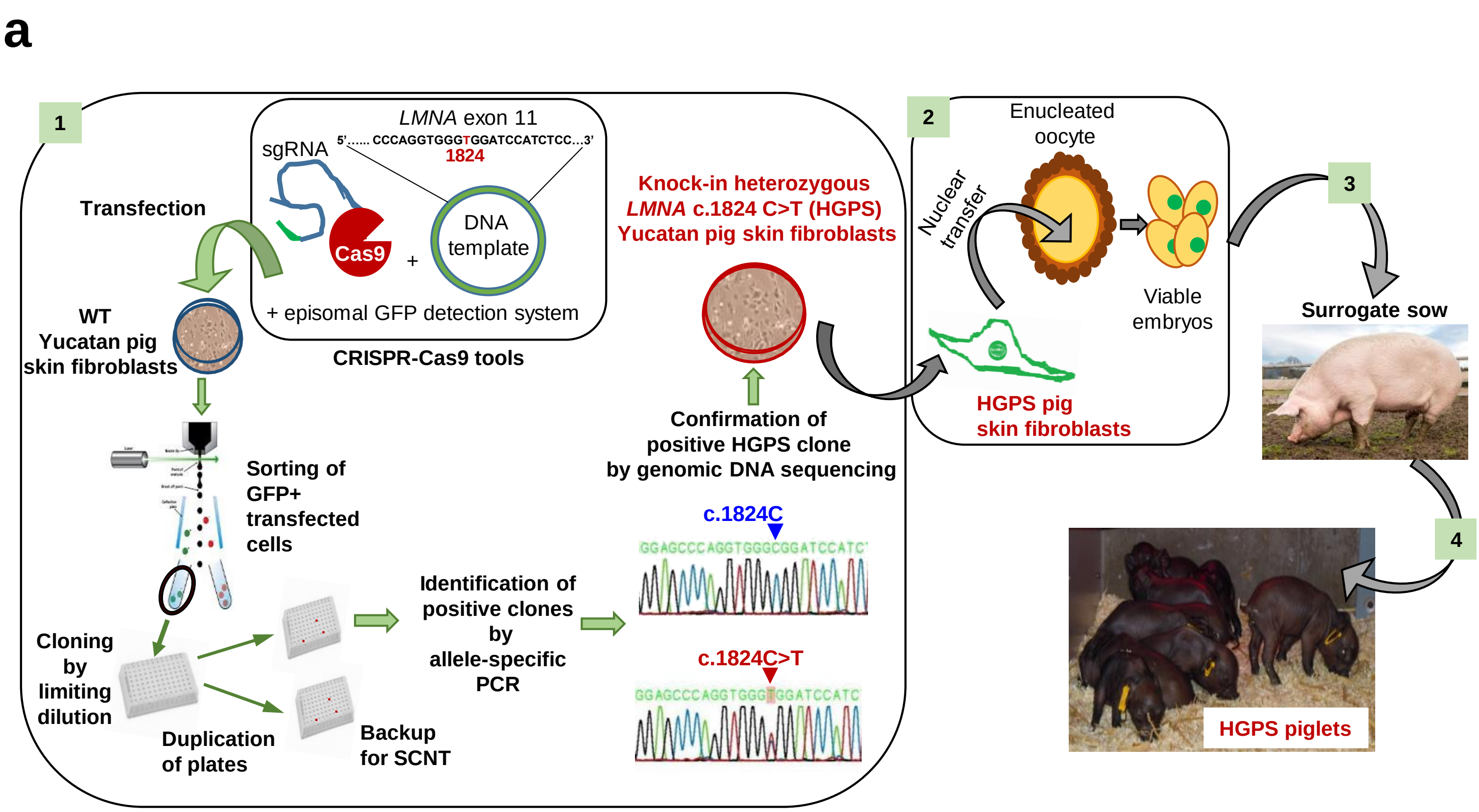

b

SCNT	Reconstructed embryos	Blastocysts/morulae	Surrogate sows	Piglets delivered
**First round cloning**	475	102 (transferred 92)	1	15 (5 stillborn, 10 liveborn -of these 3 died postnatally)
**Second round cloning**	459	137 (transferred 136)	2	4 (all liveborn -1 of these died postnatally)
				**10 HGPS piglets survived postnatal period**

Supplementary Fig. S2. Generation of knockin HGPS Yucatan minipigs.

(a) Strategy for generating knockin heterozygous *LMNA* c.1824C>T (HGPS) minipigs. (1) CRISPR/Cas9 gene editing of male Yucatan minipig primary skin fibroblasts, (2) somatic cell nuclear transfer (SCNT) into enucleated oocytes, (3) transfer of reconstructed embryos to surrogate Large White sows, and (4) pregnancy monitoring and assistance in delivery.

(b) Efficiency of the final procedures in the generation of HGPS minipigs, including SCNT of the HGPS fibroblast cell clone to enucleated oocytes and transfer of viable reconstructed embryos to surrogate sows.

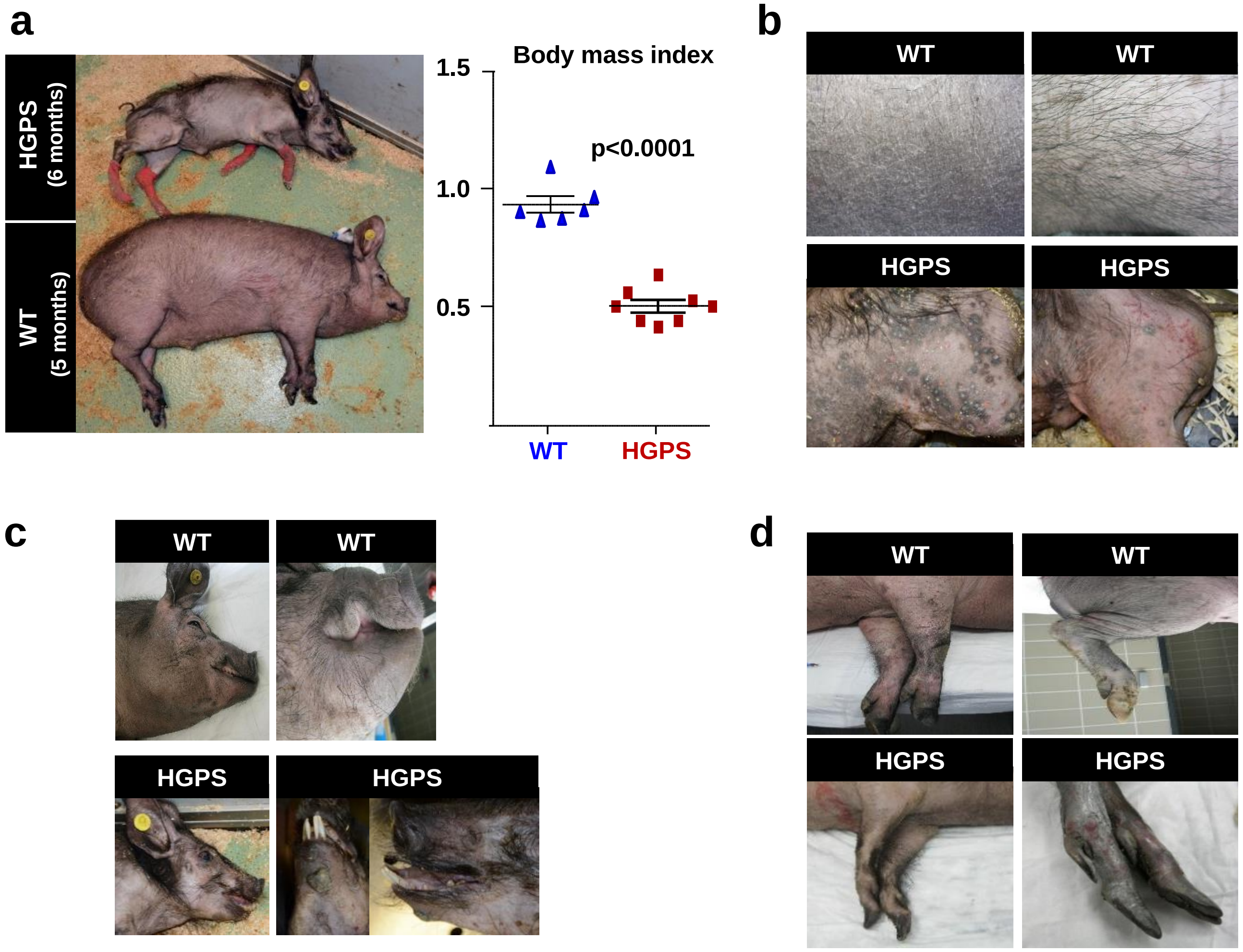

Supplementary Fig. S3. Progeroid external features in HGPS minipigs.

(**a)** Representative photographs of similar-aged HGPS and WT minipigs, illustrating the small size and lean body of HGPS minipigs. The WT minipig in this picture is a castrated male 1 month younger than the HGPS minipig. The graph shows the body-mass index of minipigs at ages between 4.3 and 5.5 months (n=6 WT; n=8 HGPS).

(b-d) HGPS minipigs recapitulate many of the external features of human HGPS, including alopecia, wrinkled and dry skin with patchy pigmentation, and sclerodermia (**b**); prominent eyes with short eyelids, sculpted nose, thin lips, and prominent teeth (**c**); as well as thin limbs with severe joint stiffness and abnormal hooves (**d**) (finger and toe alterations occur in HGPS patients).

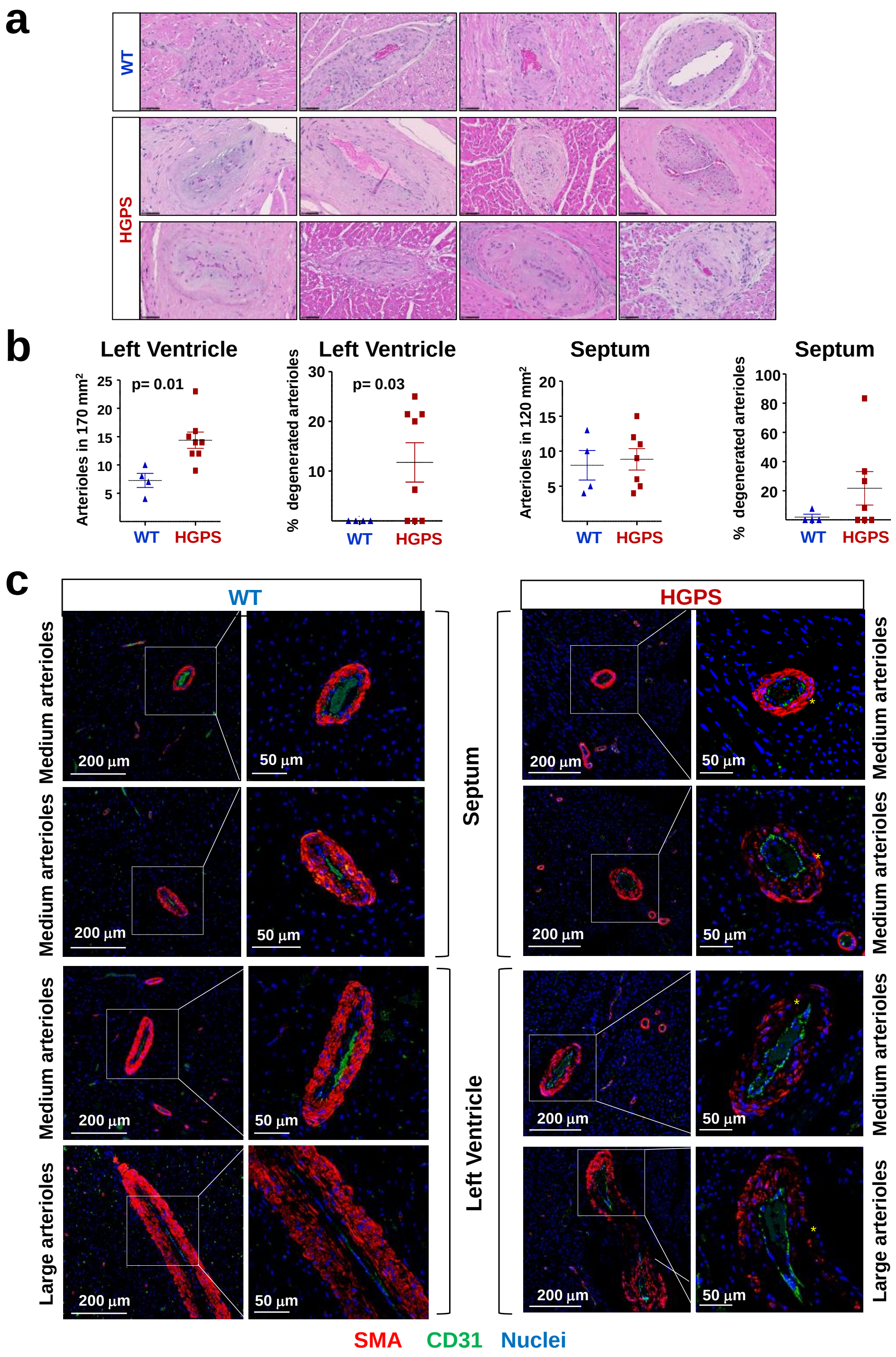

Supplementary Fig. S4. Microvascular dysfunction in the hearts of HGPS minipigs.

(a) Representative images of hematoxylin/eosin (H&E) staining in sections of medium/large LV and septal coronary arteries from WT and HGPS minipigs. Scale bar, 50 µm. **(b)** Number of arterioles >65 mm in diameter in LV (area analyzed, 170 mm^2) and septum (area analyzed, 120 mm^2), quantified by analyzing deconvoluted Masson trichrome-stained images, and the % of these arterioles showing signs of degeneration (collapsed lumen, loss of vascular smooth muscle cells, or hypertrophy in intima or adventitia). **(c)** HGPS minipigs show less expression than WT minipigs of α-smooth muscle actin (SMA) in the medial layer of LV and septal coronary arteries. Images correspond to the same experiment shown in Figure 5D, showing additional examples for LV and septum. SMA in red, CD31 (endothelial cell marker) in green, and nuclei in blue. See also **Fig. 3**.

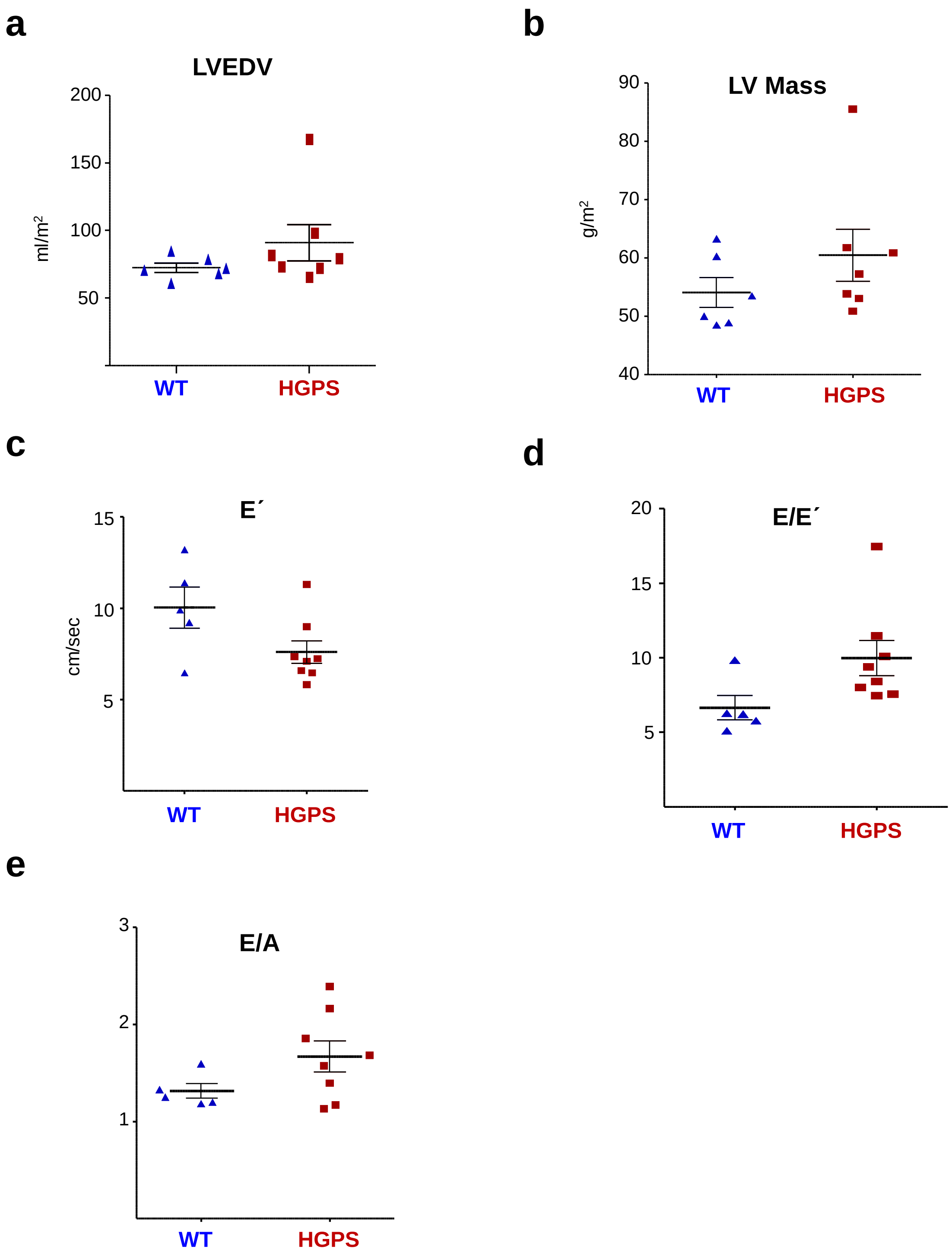

Supplementary Fig. S5. Unaffected systolic and diastolic parameters in HGPS minipigs.

Echocardiography and cardiac MRI analysis of HGPS minipigs (n=7-8) and WT minipigs (n=5-6) of a similar age (~4.3-5.5 months). **(a-b)** MRI measurements of LV end-diastolic function (LVEDV) and LV mass. **(c-e)** Echocardiographic waves.

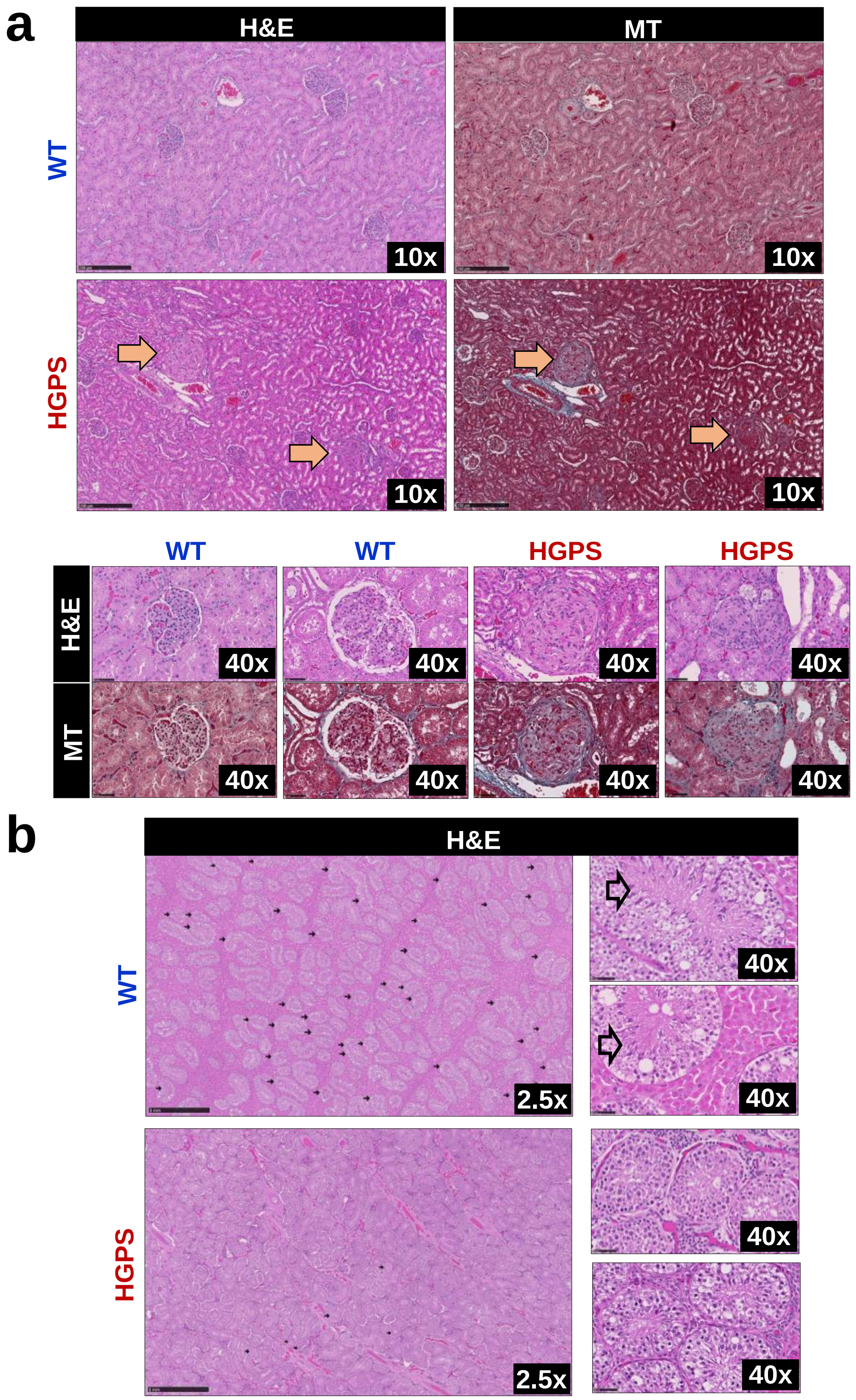

Supplementary Fig. S6. HGPS minipigs develop glomerulosclerosis and delayed testicular maturation.

(a) Representative images of hematoxylin/eosin (H&E) and Masson trichrome (MT) staining, showing dilation of cortical tubules and different degrees of glomerulosclerosis in the kidneys of HGPS minipigs. Orange arrows indicate examples of glomerulosclerosis. Scale bar, 250 µm (10x) and 50 µm (40x). **(b)** Representative images of H&E staining in testicles. Open arrows in WT indicate mature spermatids close to their release into the seminiferous tubule lumen. Solid arrows indicate tubules with elongated spermatids. Mature spermatids close to spermiation were almost absent in HGPS minipigs, which also had a reduced number of interstitial Leydig cells. Area at 2.5x is ~30 mm^2. Scale bar, 1 mm (2.5x) and 50 µm (40x).

Supplementary Table S1. Molecular tools for CRISPR-Cas9-mediated gene editing of *LMNA* exon 11 in Yucatan minipig skin fibroblasts.

Nucleotide sequences of single guide RNAs (sgRNAs), the C-check in vitro validation plasmid vector, and oligonucleotides used for generating the DNA donor plasmid.

Construct	Forward primer / oligonucleotide (5′→3′)	Reverse primer / oligonucleotide (5′→3′)
**sgRNA plasmids**		
p*LMNA*-sgRNA1 *	accgTGGGCGGATCCATCTCCTC	aaacGAGGAGATGGATCCGCCCA
p*LMNA*-sgRNA2 *	accgGCGGAGGAGCCAGAGGAGA	aaacTCTCCTCTGGCTCCTCCGC
p*LMNA*-sgRNA3 *	accgGAGATGGATCCGCCCACCT	aaacAGGTGGGCGGATCCATCTC
**p*LMNA* specific C-check plasmid ****	**GTCG**GAt(cccAGGTGGGCGGATcc aTCTCCTCtggCTCCTCCGCC)aGGT	**CGGT**ACCt(GGCGGAGGAGccaGAGGA GAtggATCCGCCCACCTggg)aTC
**p*LMNA* c.1824C>T donor plasmid**		
PCR1	CAGAACGCCACCTTCCTGCC	AGATGGATCCACCCACCTGGG
PCR2	CCCAGGTGGGTGGATCCATCT	GGTTCGGGGTCTAGAGTTGC
2-fusion PCR	CAGAACGCCACCTTCCTGCC	GGTTCGGGGTCTAGAGTTGC

* Lower case: overhang for *Bsa*I cloning; Upper case: p*LMNA* target sequence.

** Bold and underlined: overhang for *Bsa*I cloning; Lower case: PAM sites for overlapping sgRNAs; Parenthesis: p*LMNA* target region for sgRNAs.

Supplementary Table S2. Nucleotide sequences of primers used for molecular assays.

The indicated primers were used for selection of positive HGPS fibroblasts clones (c.1824C>T) by allele-specific PCR (PCR screening); for analysis of potential Cas9 off-targets (off-target analysis); for assessing random integration of undesired sequences in genomic DNA; and for genotyping of newborn piglets.

Amplicon	Forward primer 5´→3´	Reverse primer 5´→3´	Size (bp)
**PCR screening (gene targeting)**			
p*LMNA* c.1824C>T PCR	GCCTCTCAAGCCCTGTCACC	GAGCCAGAGGAGATGGATCCA	329
5´gene targeting PCR	GCCTCTCAAGCCCTGTCACC	GGTTCGGGGTCTAGAGTTGC	450
3´gene targeting PCR	CTCCCATGGCAGCAGCTCG	CCTTCCCTTGAGGTGTAGGGCAG	632
**Off target analysis**			
pChr1	CCTACCACATAGGCAGCCAGCC	CAGCGAGCCAGCAGCTGTCG	403
pChr5	GGATACATGCACAGCAAGGATTAGAG	CCACTCTGCATTCACACTGACCAG	402
pChr6-1 (*USB1*)	GGAGACTCAGGTACTTCTGGAGTGCAG	CCTTGGCCACACAGGATGTTACAG	396
pChr6-2 (*PRKCZ*)	CTGCTGCTGGCGCCATGTC	GTCTCCAGACAATTGGCCACATG	475
pChr8-1	GCCAGCCTATTGAACTGTTGGAAG	CCCACTCAGCAACAGGCTGACAC	331
pChr8-2	CAGGTTCCTAGGAGCCTGTTCCTC	GAGAGACCCTCTGGGCCATCATATC	299
pChr11	TAGATCAATGGAACAGAATAGAGAGCCC	CCTTGATGACTACTGATTTGAGCATC	612
pChr13	CTTCCTCACAGCAACATTCAATATGC	GGAACTGAAGGATTTACATTCTAGAGTGAG	508
**Random integration analysis**			
sgRNA plasmid	GGACATAAGCCTGTTCGGTTCG	ACGCCACGGAATGATGTCG	563
hCas9 plasmid	ATGGGCGGTAGGCGTGTAC	GATCTCCTGCAGGTAGCAGATC	414
EGFP-N3 plasmid	ATGGTGAGCAAGGGCGAGG	TAGTGGTCGGCGAGCTGCAC	548
Donor plasmid	GACCACCGATATGGCCAGTGTG	CCTCATCCTGTCTCTTGATCAGAGC	453
**Genotyping**			
p*LMNA* c.1824C>T PCR	GCCTCTCAAGCCCTGTCACC	GAGCCAGAGGAGATGGATCCA	329
5´ + 3´gene targeting PCR	GCCTCTCAAGCCCTGTCACC	CCTTCCCTTGAGGTGTAGGGCAG	817

Supplementary Table S3: Analysis of serum from wild-type (WT) and HGPS minipigs.

Serum samples obtained from minipigs (5.5 months old) were examined with a Dimension RxL Max analyzer (Siemens). Results are expressed as mean ± standard error of the mean (SEM). Statistical significance was calculated using unpaired two-tailed Student’s t-test.

	**WT (n=6)**	**HGPS (n=8)**	**p value**
**Triglycerides**	26.50 ± 4.288	21.13 ± 7.400	0.5771
**LDL**	33.72 ± 3.672	50.48 ± 3.211	0.005
**HDL**	57.53 ± 3.153	45.88 ± 1.441	0.0032
**Total cholesterol**	90.92 ± 5.317	103.6 ± 4.293	0.086
**Free cholesterol**	15.03 ± 1.164	19.47 ± 1.772	0.0767
**Free fatty acids**	0.4860 ± 0.05231	0.2413 ± 0.03324	0.0016
**Lipase**	22.17 ± 0.5426	32.75 ± 1.221	<0.0001
**Creatine kinase**	418.7 ± 57.14	555.0 ± 78.64	0.2141
**ALT/GPT**	49.67 ± 3.018	40.88 ± 2.539	0.0449
**AST/GOT**	30.00 ± 2.921	46.38 ± 3.391	0.0044
**Alkaline phosphatase**	94.33 ± 8.876	67.25 ± 8.998	0.0584
**C reactive protein**	0.2250 ± 0.02012	0.1188 ± 0.008952	0.0002
**Albumin**	1.650 ± 0.06191	1.413 ± 0.07181	0.0337
**Total proteins**	7.217 ± 0.1579	6.525 ± 0.09210	0.0017
**Creatinine**	0.9100 ± 0.07488	0.9175 ± 0.09248	0.9533
**Urea nitrogen**	12.17 ± 0.6540	21.88 ± 1.737	0.0006
**Direct bilirubin**	0.0400 ± 0.007303	0.02125 ± 0.003981	0.0328
**Total bilirubin**	0.5833 ± 0.02333	0.1525 ± 0.02358	<0.001
**Glucose**	78.83 ± 4.301	105.9 ± 15.38	0.1656
**Potassium**	4.050 ± 0.1176	4.613 ± 0.1575	0.0198
**Calcium**	10.47 ± 0.1706	10.50 ± 0.1268	0.8751
**Sodium**	134.8 ± 0.7491	139.8 ± 1.719	0.0377
**Phosphorus**	9.200 ± 0.1653	8.287 ± 0.1941	0.0051

SUPPLEMENTARY MOVIES

Supplementary Movie S1. Normal appearance and behavior of newborn HGPS piglets, Related to Figures 1 and 2. The movie shows a foster Large White sow nursing HGPS Yucatan piglets (13 days old, black skin) and a wild-type Large White piglet (pink skin). The wild-type piglet was introduced into the litter after farrowing to maintain milk production. The HGPS piglets have a normal appearance and behavior, with no apparent progeroid symptoms.

Supplementary Movie S2. First symptoms of joint stiffness and abnormal walking in HGPS pigs, Related to Fig. 1 and Fig. S3. HGPS minipigs of ≈2-2.5 months of age showing light stiffening of the legs and abnormal walking.

Supplementary Movie S3. First symptoms of joint stiffness and abnormal walking in HGPS pigs, Related to Fig. 1 and Supplementary Fig. S3. HGPS minipigs of ≈3 months of age showing light stiffening of the legs and abnormal walking, and first changes of facial appearance.

Supplementary Movie S4. Abnormal movement and development of external progeroid features in HGPS minipigs aged 5 months, Related to Fig.1 and Supplementary Fig. S3. The movie illustrates the development of stiffness of the carpal and tarsal joints and at the phalanx level in HGPS minipigs aged 5 months, with abnormal movement more evident compared with younger HGPS minipigs. Nevertheless, the animals were able to feed autonomously and interacted with their environment.

Supplementary Movie S5. Normal appearance and movements in a wild-type Yucatan minipig (4 months old), Related to Fig. 1 and Fig. S3. Occasionally, 5 months old HGPS Yucatan minipigs are seen in an independent pen on the right.
